# The mouse that trolled: the long and tortuous history of a gene mutation patent that became an expensive impediment to Alzheimer's research

**DOI:** 10.1093/jlb/lsv011

**Published:** 2015-04-23

**Authors:** Tania Bubela, Saurabh Vishnubhakat, Robert Cook-Deegan

**Affiliations:** 1 School of Public Health, University of Alberta, Canada; 2 School of Law, Duke University, USA; 3 Sanford School of Public Policy, Duke University, USA

**Keywords:** Alzheimer's disease genetics, research tool, non-practicing entity, patent litigation, patent troll reform, enablement, Authorization and Consent

## Abstract

This case study presents the tale of the academic discovery of a rare mutation for early-onset Alzheimer's disease that was patented by a sole inventor and licensed to a non-practicing entity (NPE), the Alzheimer's Institute of America (AIA). Our aims are (1) to relate this story about patents, research tools, and impediments to medical progress, and (2) to inform ongoing debates about how patents affect research, disposition of university inventions, and the distribution of benefits from publicly funded research. We present an account of the hunt for Alzheimer's genes, their patenting, assignment, and enforcement based on literature, litigation records and judicial decisions. While AIA's litigation eventually failed, its suits against 18 defendants, including one university, one foundation, and three non-profit organizations were costly in court years, legal fees, and expert time. Reasons for the failure included non-disclosure of co-inventors, State laws on ownership and assignment of university inventions, and enablement. We discuss the policy implications of the litigation, questioning the value of patents in the research ecosystem and the role of NPEs (“patent trolls”) in biotechnological innovation. The case illustrates tactics that may be deployed against NPEs, including, avenues to invalidate patent claims, Authorization and Consent, legislative reforms specifically targeting NPEs, reforms in the America Invents Act, and judicial action and rules for judicial proceedings. In the highly competitive research environment of Alzheimer's genetics in the 1990s, patents played a minor, subordinate role in spurring innovation. The case produces a mixed message about the patent system. It illustrates many mistakes in how patents were obtained, administered, and enforced, but, eventually, the legal system rectified these mistakes, albeit slowly, laboriously, and at great cost.

This case study is a cautionary tale about a patented genetic discovery, a double mutation in a gene conferring high risk of Alzheimer's disease. The patent was licensed as a research tool to expand understanding of the molecular and genetic basis of Alzheimer's disease. Our goals are first, to relate an intricate, fascinating story about patents, research tools, and impediments to medical progress and, second, to inform ongoing debates about whether and how patents can affect research, disposition of ownership of university inventions, and the just distribution of benefits from publicly funded medical research.

Randall Rader, in his dissent as former Chief Judge of the US Court of Appeals for the Federal Circuit (CAFC) in *Momenta Pharma. v Amphastar Pharma*, concluded that ‘patents on research tools and biomedical innovations do not significantly slow the pace of research and do not deter researchers from pursuing promising projects’.^1^ Patent owners, he said, do not sue researchers because ‘experiments do not produce income or a source of damages’.^2^ Indeed, disclosure in patent documents ‘encourages publication and sharing of research results’.^3^ Our case study is a counterexample to temper Chief Judge Rader's sanguine assessment. While it describes an unusual outlier example and is not an argument against patents, this case study suggests that without a clear research exemption, or other mechanisms to enable access to research tools, biomedical researchers can face patent infringement litigation that imposes significant costs and slows down both academic and commercial scientific inquiry.

The story began in the early 1980s with research into the genetics of Amyloid Precursor Protein (APP) and its linkage to early-onset Alzheimer's disease. Key researchers were active in Europe and the USA. Patents on a particular double mutation, *APPswe*, were filed in the early 1990s, listing a clinician-researcher, Dr Michael Mullan, as sole inventor. The patents covered nucleotide sequences coding for a rare double mutation identified in two Swedish families. The patents also claimed cell lines, transgenic mouse models, and assay and screening methods incorporating the mutation. The institutions that hosted the research were deliberately excluded from the patent rights. Stakeholders affected by the patents included other researchers and their institutions, pharmaceutical and biotechnology companies, philanthropists, foundations, and venture capital firms. The lead researcher, Mullan, assigned key patents to a non-practicing entity, the Alzheimer's Institute of America (AIA), which enforced rights against research uses. The patent rights were used to generate revenues, but the disposition of those revenues is difficult to trace. AIA may have sponsored some of Mullan's further research but is not acknowledged as a funder in the research publications from Mullan's team.^4^ The families from whom the mutations were isolated received none of the financial benefits, and are likely unaware that their mutation was used to enrich one researcher and a venture capitalist with the result of impeding Alzheimer's research. Ironically, after nearly a decade of litigation, claims in the patents might or might not have been deemed valid under current patent jurisprudence.^5^ We will never know, because the patents were deemed invalid based on inappropriate assignment and inventorship.

The case study is of interest because it illustrates a non-practicing entity (NPE) using patent rights over research tools to extract revenue from those striving to understand and treat Alzheimer's disease. AIA's enforcement against non-profit research institutions caused considerable consternation in Alzheimer's research circles. The case study touches on key points in ongoing debates about the value of patents in the research ecosystem. Did patent rights create incentives to do the research? Almost certainly not, at least for the initial discovery, since the samples were collected and grants secured long before the patent story began to unfold. How were contributions to the research evaluated and rewarded? How did legal frameworks enable university ownership, even without federal funding in the USA? How was enforcement of patent rights against non-profit research institutions enabled, and who was benefited? What mechanisms were brought to bear to mitigate the impact of the enforcement litigation? The case illustrates many mistakes in how patents were granted, administered, and enforced, but, in the end, the legal system rectified many of these errors, albeit after long delay and at great expense.

We begin with a brief account of the genetics of early-onset familial Alzheimer's disease and the discovery of the *APP* gene, then move on to the patents, and finally discuss patent assignment and enforcement: the resolution of many infringement lawsuits, and the lessons learned. Our account is based on data available in the public record from disparate sources: scientific publications, patents, news and commentary, biographies, and most importantly, litigation records and judicial decisions. The legal proceedings include findings of fact by the district court judge and/or jury based on the evidence presented. The role of the trial judge (and the jury in some details) is to assess the credibility of documentary evidence and oral testimony. This assessment leads to an accounting of what ‘really’ happened, even though in litigation, there are by definition multiple sides to the story.^6^ Appendix 1 presents a graphic chronology of the key events in the story.

## ALZHEIMER'S DISEASE: FACTS AND FIGURES

Late Onset Alzheimer's Disease (LOAD) is the most common form of dementia.^7^ By 2025, it is expected that 7.1 million people over 65 years of age will be affected in the USA. The health stakes are high, and so is the prospect of profit from effective drugs to treat or prevent Alzheimer's disease.^8^ The associated economic burden for patients, their families, and society is staggering; current costs to the healthcare system exceed ${\$ }$200 billion annually.^9^ LOAD is caused by a combination of genetic and environmental risk factors. There are at least ten genes that account for half of the genetic risk for Alzheimer's, including variants of the APOE gene.^10^ The ε4 allele of APOE has the strongest association with LOAD,^11^ the physiological features of which are the accumulation of the protein fragment beta-amyloid (plaques) in the brain and tangles inside neurons made up of the protein tau.

Early Onset Alzheimer's Disease (EOAD), on the other hand, accounts for only a few per cent of cases and begins before the age of 65.^12^ EOAD runs in families with an autosomal dominant inheritance pattern and is caused by one or more mutations on any of the genes for: the amyloid precursor protein (*APP* gene, chromosome 21), the presenilin 1 protein (*PSEN1* gene, chromosome 14), or the presenilin 2 protein (*PSEN2* gene, chromosome 1). Mutations in these genes cause the disease and account for 16, 66, and 18 per cent of early-onset cases, respectively.^13^ The race to identify these genes is one focus of this paper.

Alzheimer's disease was first described in 1906 by German physician Dr Alois Alzheimer.^14^ The dominant explanation for its cause is the amyloid cascade hypothesis.^15^ This hypothesis suggests that the central event in Alzheimer's disease pathology is the deposition in the brain of amyloid-β, a fragment of a transmembrane protein, APP. Nevertheless, the correlation between dementia or other cognitive alterations and amyloid-β accumulation in the brain in the form of amyloid plaques is not linear, and mutations in multiple genes are likely involved.^16^

Despite decades of research and billions of dollars of investment, no therapies have been approved by regulatory agencies that slow or stop the course of either the early- or late-onset forms. Approved therapies, of which there are five, ameliorate symptoms in some patients.^17^ In 2013, there were 65 clinical trials for new therapeutic approaches, the vast majority in early phases I and II.^18^

## HISTORICAL BACKGROUND

### Hunt for the first gene for EOAD

The field of Alzheimer's genetics during the heyday of gene hunting was highly competitive, described as ‘a mixture of idealism, selfishness, generosity, greed, fun, anger, sex, drugs and rock ‘n’ roll’.^19^ The key prize for the competitive teams was high-impact publications, predominantly in *Science* and *Nature*. According to those engaged in the races for the Alzheimer's genes, patents were secondary to scientific priority. The prospect of commercial gain was considered, but not dominant. Autobiographies from the era describe shifting allegiances and collaborations among research groups, especially with respect to the biological samples shared.^20^ Some researchers had close relationships with members of the families studied and their clinicians.^21^

In 1984, Dr John A. Hardy headed a research team at St Mary's Hospital Medical School in London that was searching for the genetic basis of Alzheimer's disease. In 1988, St Mary's Hospital merged with Imperial College, at which time Michael Mullan joined Prof. Hardy's team as a Clinical Research Fellow. He received a Ph.D. in 1993. The Hardy team was one of many, internationally, that was seeking the gene(s) responsible for EOAD. Also in the hunt were multiple groups in the USA, Europe, Japan, and Australia. This research depended on clinical diagnosis of Alzheimer's disease, biological samples from the patients and other family members, and detailed pedigrees.^22^ In pedigrees of 4000 known descendants with sixty known cases of the disease, it was clear that EOAD was caused by a genetic mutation.

Two key lines of evidence directed the hunt toward chromosome 21. First, individuals with Down syndrome, caused by an extra copy of chromosome 21, accumulate an amyloid protein similar in structure to individuals with Alzheimer's disease.^23^ In addition, Rudolph Tanzi^24^ was constructing a linkage map of chromosome 21, making it a particularly inviting target for etiological research.^25^ Despite the evidence, however, initial testing of linkage of EOAD to markers for Down syndrome was negative.^26^ But, further testing in some families^27^ showed a genetic linkage to a different region that contained the *APP* gene.^28^ In 1987, four papers were published almost simultaneously that mapped the *APP* gene to chromosome 21 and sequenced portions^29^ or all of the gene.^30^ Unfortunately, later that year, researchers found no linkage between any of the Alzheimer's families and the *APP* gene.^31^ Thus, the hope for a quick answer for the cause of Alzheimer's in the spring rapidly began to evaporate by September of the same year.^32^

At the same time, researchers at the University of Leiden in the Netherlands had shared samples of families with a rare condition known as the Dutch disease.^33^ In 1990, two groups linked the disease to the *APP* gene.^34^ This evidence inspired Hardy's team to check its EOAD samples for mutations in the same region on chromosome 21. His team found a mutation close to the Dutch disease mutation^35^ in samples from a small British family^36^, and the same mutation in a second family's samples that they received from Dr Allen Roses,^37^ samples on which Dr Roses had found a linkage to chromosome 21. However, no samples from the other 22 families kept by Hardy's group carried the mutation. His team published this first Alzheimer's mutation in the *APP* gene in *Nature*.^38^ This paper was the first to link a genetic mutation with the molecular basis of Alzheimer's disease and became the most cited publication in the biomedical literature in 1991.^39^ The paper was not merely famous, but infamous as well, causing great controversy in the Alzheimer's research community for omitting Roses as a co-author.^40^ Science Watch labeled research on the *APP* gene as the ‘hottest corner of biology’.^41^ The mutation became known as the ‘London mutation’.

After the publication of the London mutation, teams around the world looked for the mutation in their EOAD families. One was found in France and three in Japan, but the mutation was obviously rare.^42^ Indeed, in the end it became obvious that the initial publication by St George-Hyslop claiming linkage to chromosome 21 was wrong. As explained by Tanzi in *Decoding Darkness*:

because of Hyslop's finding that hinted at something amiss on chromosome 21, John Hardy's lab had determined it too had a family linked to chromosome 21. Yet while the original [Massachusetts General Hospital] data turned out to be false ^43^, the Hardy team's linkage to chromosome 21 was the originally suspected *APP* gene! “It was a bizarre conundrum that the original linkage report from Mass General was wrong”. Recounts John Hardy, “Bizarre, because it was wrong in the right place”. *^44^*

### Commercialization of the London mutation

At this point in the narrative, issues of commercialization begin to enter the story, setting the stage for later discovery of the Swedish (*APPswe*) mutation. The findings of fact in the litigation start at this point, with the discovery of the London mutation.^45^ Judge Savage of the US District Court for the Eastern District of Pennsylvania outlined the history of the London mutation because this negative experience impacted Hardy and Mullan's subsequent commercialization endeavors.^46^

In January 1992, the Imperial College of Science, Technology and Medicine filed for a US patent that claimed the nucleic acid sequence encoding a codon 717 mutant of human APP 770 and associated cDNA and cell lines.^47^ The patent listed Hardy and Mullan, along with Marie-Christine Chartier-Harlin, Alison Goate, and Michael Owen as inventors. According to Judge Savage, technology transfer officers at Imperial Exploitation Limited (‘IMPEL’) erroneously advised the team that UK law prohibited the patenting of transgenic animals and, so the patent did not claim transgenic animal models.

After the filing, Athena Neurosciences, a San Francisco-based biotechnology company, approached Imperial, through Hardy, ‘to sponsor the team's research on new APP mutations’.^48^ This resulted in a Sponsored Research Agreement in August 1991. ‘Athena then redrafted the US patent application to include transgenic animals carrying the London mutation. The agreement granted Athena exclusive rights to mutation-carrying transgenic animals and to any subsequent Alzheimer's discoveries from the laboratory. Upon learning that IMPEL had given them erroneous advice, the research team was disappointed with the deal they had made with Athena.’^49^ Imperial rejected Hardy and Mullan's attempt to renegotiate their interest in the patent because under the *U.K. Patents Act, 1977*, inventions made during the normal course of employment vested in the employer, not the employee.^50^

The dispute with Imperial over the patenting and licensing of the London mutation was part of the impetus for the researchers to leave Imperial, and warm to recruitment by the University of South Florida (USF), which was enticing them with financial and research incentives. Ronald Sexton, a Kansas City businessman and venture capitalist who became central to the ensuing litigation over the Swedish mutation, actively encouraged the move. By the end of 1991, Mullan had relocated to Florida to set up the Alzheimer's research laboratory to be headed by Hardy; his employment there began on December 16, 1991. Mullan later claimed that the move was due to the poor funding environment for Alzheimer's research in the UK, even though USF was ‘not necessarily one of the top places’.^51^

## DISCOVERY OF THE SWEDISH MUTATION AND THE KEY PROTAGONISTS

The discovery of the London mutation triggered other researchers to screen their samples for mutations in the region of Exon 17. Dr Lars Lannfelt, then working with Dr Bengt Winblad at the Karolinska Institute in Sweden, was collecting samples from Swedish families with a hereditary pattern of EOAD. In February 1992, Lannfelt visited Hardy in London with pedigrees and samples from two of his families. Hardy instructed one of his students, Henry Houlden, to test the DNA for linkage against marker GT12 near APP on chromosome 21. The linkage analysis found a strong likelihood of a mutation on the *APP* gene in both Swedish families.^52^ Hardy then sent the samples of the affected and unaffected members of the Swedish families to Mullan for sequencing in Florida to check for mutations on Exons 16 and 17.^53^ This action contradicted Lannfelt's understanding, stated in an interview, that the sequencing was to be divided between the teams, with Exon 16 to be sequenced in Florida and Exon 17 in Sweden.^54^

When the samples arrived in Florida, Mullan confirmed the linkage. Without consulting Hardy, Mullan instructed Fiona Crawford, another member of the Hardy team who had moved to Florida, to sequence Exons 16 and 17 ‘at an off-campus laboratory at the Tampa Bay Research Institute (‘TBRI’) instead of in the USF laboratory’.^55^ The sequencing confirmed a double *APP* gene mutation affecting codons 670 and 671. The results were published in *Nature Genetics* in 1992 with Mullan as the corresponding author using his USF affiliation.^56^ Also included among the authors on the paper were Winblad and Lannfelt from the Karolinska Institute, Houlden from Imperial College, and Fiona Crawford from USF. Notably absent as co-author (and even from the acknowledgements), however, was Hardy, because Mullan and Hardy ‘agreed that Hardy's name would not be included on any publication related to the Swedish mutation’.^57^ In addition, the only funding sources acknowledged on the paper are Swedish: the Swedish Medical Research Council and the Tore Nilson Fund. Other publications from the Mullan-Hardy team between 1991 and 1993 cited support from the Medical Research Council, the Wellcome Trust, and a variety of disease charities, among others.^58^

According to the judge and jury that sifted through trial documents and testimony, the sequencing was structured deliberately to avoid ownership claims by Imperial College.^59^ Indeed, Judge Savage concluded: ‘Having the sequencing done in Florida rather than in London could have been seen by the jury as nothing more than a step in the furtherance of the conspiracy to avoid the inventions becoming the property of Imperial College or Athena’.^60^ Leaving Hardy's name off the publications seems consistent with this conclusion as well. At the time, Hardy was still employed at Imperial College, and both Mullan and Crawford were still Ph.D. students.^61^ Imperial College, like most universities, required disclosures of inventions by staff and students; it also required assignment of ownership rights.^62^ Such disclosure and assignment were not made. Indeed, Mullan claimed that he was not a student at Imperial College in 1992 although he was still working on his dissertation at that time.^63^

In 1992, Mullan, Hardy, and Sexton engaged a UK law firm, Clyde & Co., to draft a letter to USF asking for a waiver of any rights USF may have to any ‘inventions made by them, whether before or after the date of the letter’, which would include any rights in the Swedish mutation.^64^ The USF President of Research, Dr George R. Newkome, signed the letter on May 4, 1992, but changed the language to ‘before August 15, 1992’. However, the letter did not specifically make reference to the Swedish mutation, which was discovered while Mullan was already employed at USF, and USF's knowledge of this discovery was disputed years later, during the litigation.

A day after the letter was signed by all parties, Sexton incorporated the AIA as a Florida for-profit corporation on May 5, 1992, for ‘the purpose of holding and exploiting the rights to the Swedish mutation’.^65^ AIA issued its first annual report in 1993, naming Sexton as President and Mullan as Vice President and treasurer.

Following the publication on the Swedish mutation, Mullan sought US patent protection as sole inventor (see Table 1). The first patent application was filed in June 1992 and a patent issued in October 1995.^66^ A second patent application, also naming Mullan as sole inventor, was filed in March 1997 and granted in August 1998, claiming the polypeptide of the APP protein with the Swedish mutation (Table 1). Finally, in March 2007, Mullan filed another continuation application claiming transgenic mice that contained the *APPswe* mutation, again as sole inventor. That third patent was granted in May 2009, fully 17 years after the initial patent application was filed.^67^ The third patent claimed transgenic mice carrying the Swedish mutation as well as screening methods for an Alzheimer's therapeutic agent using such mice (see Table 1). Mullan assigned his rights in these patents to AIA.

**Table 1.** AIA litigation related patents naming Michael Mullan as inventor and AIA as assignee. Note that all patents claimed the June 4, 1992 priority date.

Patent number	Title	Subject matter	Filed/Published
US 5,455,169	Nucleic acids for diagnosing and modeling Alzheimer's disease	Claims (1) the nucleic acid of the human APP with asparagine at codon 670 and/or leucine at codon 671, or a fragment of the protein; (2) a further specification about the amino acid at codon 717; (3) a vector that includes the nucleic acid; and (4) an immortalized mammalian cell line that contains the nucleic acid in question.	1992-06-04/1995-10-03
US 5,795,963	APP in Alzheimer's disease	Claims the purified and isolated polypeptide of human APP that includes codons 670 and 671, where asparagine is at codon 670 and/or leucine is at codon 671.	1997-03-13/1998-08-18
			
US 6,818,448	Isolated cell comprising HAPP 670/671 DNAS sequences	Claims (1) an isolated (and immortalized) cell with a nucleic acid encoding a human APP that includes codons 670 and 671, operable linked to a promoter. The nucleic acid encodes an amino acid other than lysine at codon 670 and/or an amino acid other than methionine at codon 671. The cell expresses the human APP or a fragment of it; (2) the same claim that includes codon 717, with an amino acid other than valine; and (3) a method of screening for an agent for treating Alzheimer's disease that involves contacting the claimed cell with an agent and monitoring the expression or processing of APP or fragments thereof.	2001-02-16/2004-11-16
US 7,538,258	Transgenic mouse expressing an APP 670/671 mutation	Claims (1a) a transgenic mouse whose genome comprises a nucleic acid encoding human APP including codons 670 and 671, operably linked to a promoter, where the amino acid at codon 670 is not lysine and/or the amino acid at 671 is not methionine and the mouse expresses this protein or a fragment of it; (b) a transgenic mouse that has an amino acid other than valine at codon 717; and (c) the mouse forms amyloidogenic aggregates in its brain and/or exhibits Alzheimer's disease pathology; and (2) a method of screening for an agent for treating Alzheimer's disease through contacting the mouse with an agent and monitoring the expression, processing or deposition of APP ,or fragments of it.	2007-03-08/2009-05-26

In 1993, the relationship between Hardy and Mullan became strained.^68^ In *Decoding Darkness*, Tanzi quoted Mullan as stating that he had ‘privately patented’ the Swedish mutation ‘because I'd found it, and because [Hardy] hadn't been convinced the Swedish family even had a mutation’.^69^ According to Tanzi, Hardy inadvertently came across correspondence between Mullan and a California biotechnology company about commercially available mice with the Swedish mutation. While Mullan disputed that the correspondence was about the Swedish mutation, commercialization endeavours and the US research environment drove a wedge between Hardy and Mullan^70^ to the point that Hardy became a key defense witness against Mullan's patents in later litigation.

Within a decade, both Hardy and Mullan had left the Department of Psychiatry at USF. Hardy accepted a position in 1997 at the Mayo Clinic in Jacksonville, Florida, where he collaborated with SmithKline Beecham and the Institute for Genome Research.^71^ Mullan moved to the Roskamp Institute, off campus in Sarasota, the home of Robert Roskamp, a ‘businessman and USF benefactor who contributed ${\$ }$5 million to set up the center’.^72^ Mullan resigned from USF in January 2003 ‘after a USF investigation concluded that he had sexually harassed one woman and created ‘a serious risk’ of violating university policies in his pursuit of personal relationships with women in his lab’.^73^ *Nature News* reported that Mullan, after resigning from USF, filed a civil lawsuit for defamation against the former USF researcher he had allegedly harassed in 1997.^74^

The Roskamp Institute was set up as a non-profit organization in 2003, and its website stated that it was affiliated with the AIA.^75^ Until 2003, the Roskamp Institute was part of the USF, and in 2009, Mullan became its executive director; it spun out Archer Pharmaceuticals in 2008 with Mullan as chief executive officer and chief scientific officer, and with Fiona Crawford as associate chief scientific officer.^76^ The company specialized in targeted drug discovery for Alzheimer's disease. Archer Pharmaceuticals was also listed as a collaborator in early trials of nilvadipine in Europe that Trinity College led, beginning in 2006.^77^

At Roskamp, Mullan continued his relationship with Ronald Sexton and the AIA, located in Kansas City, Kansas, as well as with its affiliated charitable AIA Foundation located in Sarasota, Florida. These two entities had similar names, with one for-profit and the other non-profit. The Directors of AIA were listed as Sexton and Marjorie E. Curran. Those of the non-profit AIA Foundation, founded in 2010,^78^ were listed as Michael Mullan, Brian Sexton, and Jamison Sexton (now at Roche Diagnostics). According to separate websites for both entities, partnering organizations included the Roskamp Institute, the Green-Field Library, and Archer Pharmaceuticals prior to May 29, 2011. The AIA website listed the Swedish mutation patents among its holdings, but contained no information on commercial or academic licensing opportunities. The current website links for licensing the *APPswe* patents from AIA are inactive.^79^

### Summary of research on the *APP* gene

**Figure 1.**
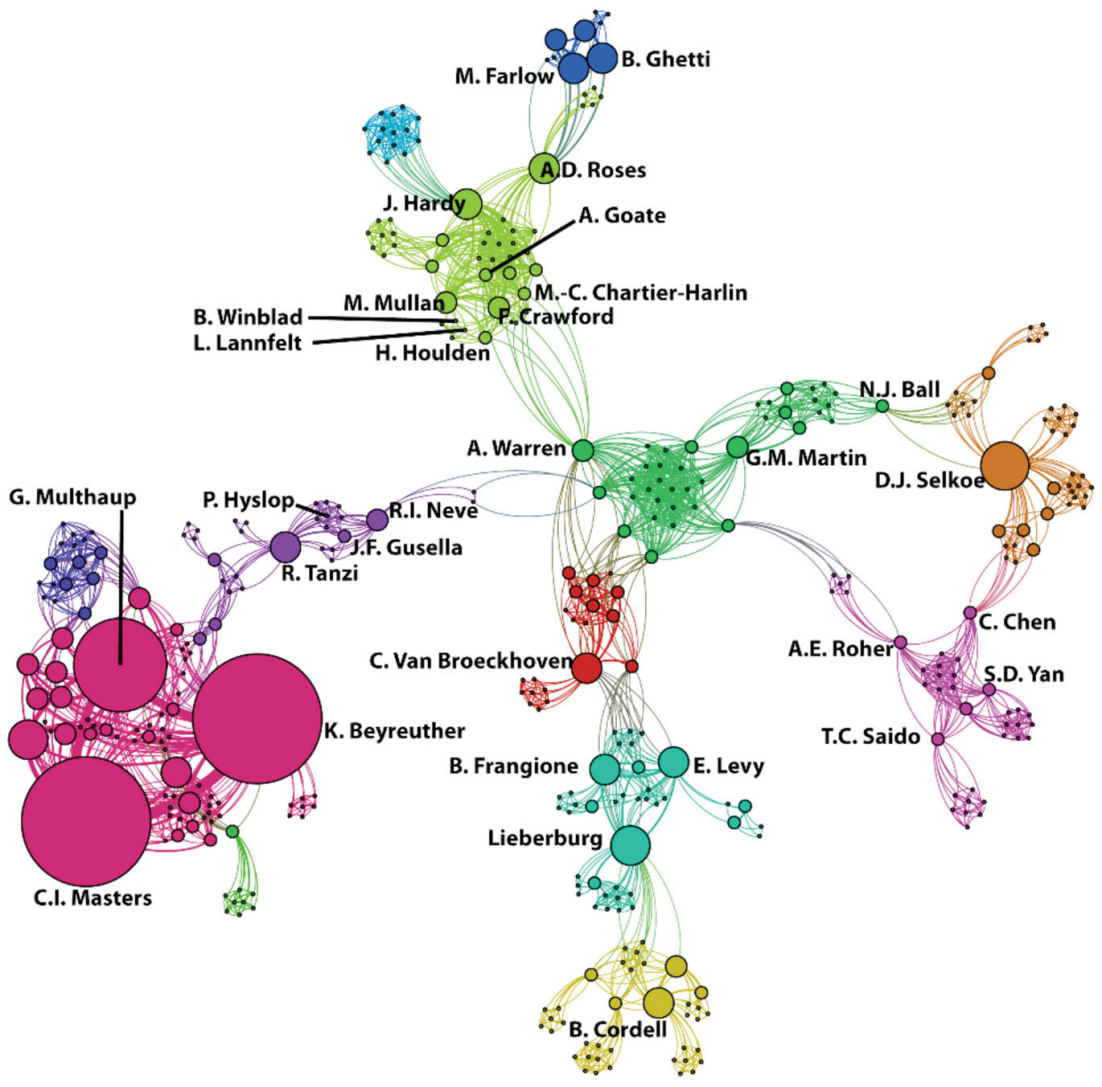
Co-authorship of 145 publications on the *APP* gene prior to 2000.^80^ Colors indicate clusters of individuals who published together. Linkages (co-authorship) between authors are indicated with lines. The size of the circle indicates the relative number of publications attributed to an author. We added author names for those who (1) had a large number of papers, (2) were central in linking two groups, (3) were illustrative of a grouping, and/or (4) were central actors in patenting mutations in the *APP* gene.

**Figure 2.**
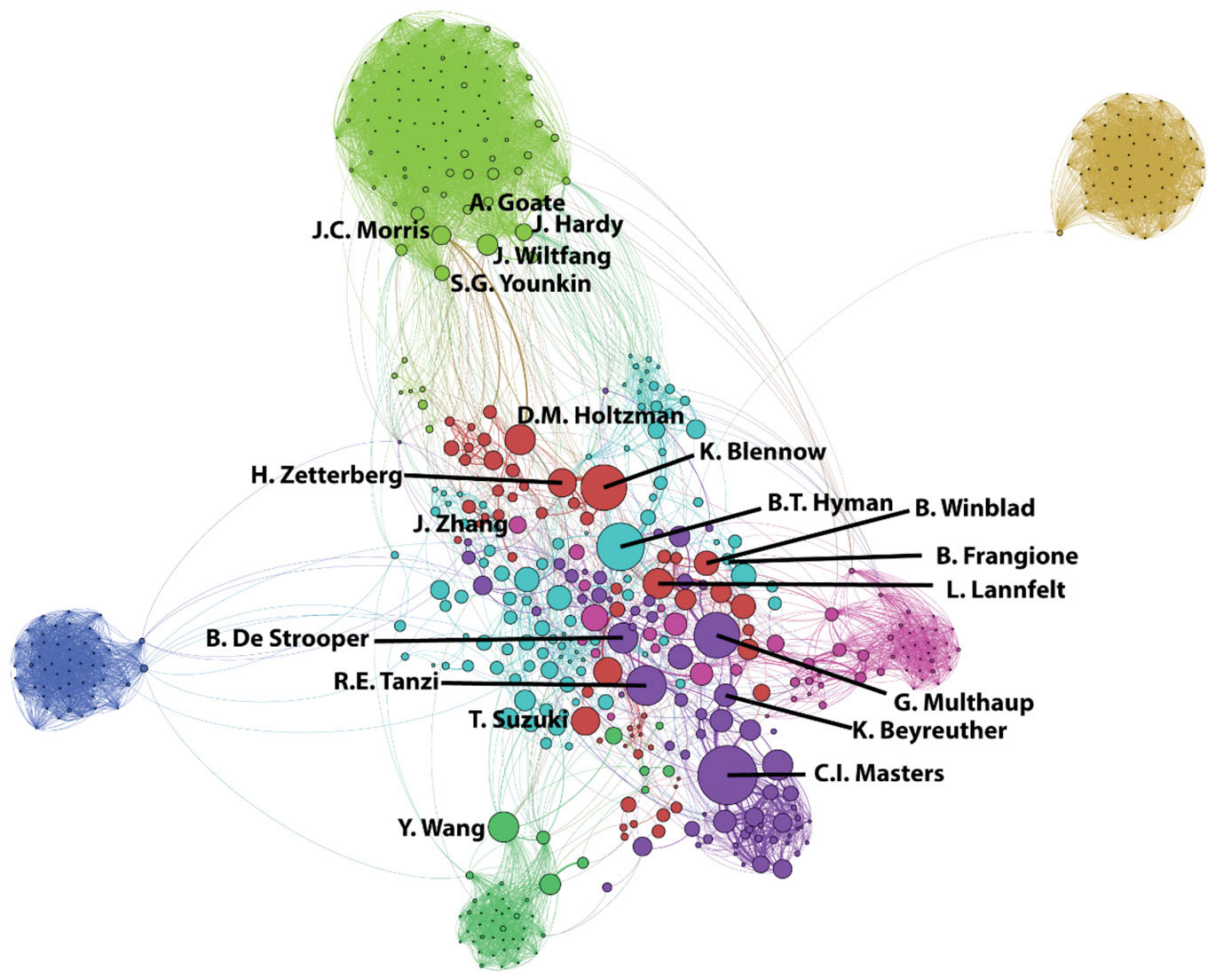
Co-authorship of 1724 publications on the *APP* gene from 2000 to 2014.^81^ Colors indicate clusters of individuals who published together. Linkages (co-authorship) between authors are indicated with lines. The size of the circle indicates the relative number of publications attributed to an author. We added author names for those who (1) had a large number of papers, (2) were central in linking two groups; (3) were illustrative of a grouping, and/or (4) were central actors in patenting mutations in the *APP* gene.

The history of research on the *APP* gene and the relative contributions over time to the field are illustrated in Figures 1 and 2. Figure 1 shows linkages by co-authorship of publication on the *APP* gene up to the end of the 1990s, with the bulk of research papers on the genes generated by researchers at the University of Heidelberg, Germany (the Konrad Beyreuther— Gerhard Multhaup—Colin Masters cluster). Other central groups are led by Frangione, van Broeckhoven, and Hardy. Hardy's group includes his students—Mullan, Crawford, and Houlden—and links to Dr Alan Roses and two Swedish researchers, Drs Bengt Winblad and Lars Lannfelt. The Gusella group includes St George-Hyslop and Tanzi. The research communities (differentiated by color) in this graph have limited connectivity, illustrating the competition between the separate teams in the 1990s that were racing to find and characterize mutations on the *APP* gene.

In the 2000s (Figure 2), the field became more widely dispersed. Notably, there is far more connectivity among the research communities. Colin Masters’ research continued in Australia. Masters also continued collaboration with the German researchers Beyreuther and Multhaup. The Swedish researchers, Lannfelt and Winblad, increased their publications in the field. Others from the 1990s, such as Tanzi and Frangione, continued to publish. New clusters of researchers entered the field. Hardy, in collaboration with Goate, continued with a smaller number of publications on the *APP* gene. However, the researcher who claimed a sole proprietary interest over one key mutation, Michael Mullan, is notably absent as a co-author on any paper, although he continued in the field of Alzheimer's research.

## THE LITIGATION

The patent lawsuits over Alzheimer's research, in general, and the Swedish mutation, in particular, run against perceptions that publicly funded researchers are immune from patent infringement litigation. The central enforcer was AIA, an NPE created to exploit the Swedish mutation patents. Defendants included pharmaceutical and biotechnology companies as well as public and private research institutions. In five lawsuits, AIA asserted its patents against 18 separate defendants, including one university, one foundation, and three non-profit research organizations. The litigation consumed at least 18.7 cumulative court years in six jurisdictions; engaged at least 98 lawyers on the record (39 engaged by non-profit research organizations, foundations, and universities); and entailed 1143 court filings (docket entries) for motions, pleadings, complaints, certifications, demands, notices, affidavits, stipulations, etc. (Table 2).^82^ AIA was not successful in any litigation; all cases were dismissed with no dispositive finding of patent infringement (Table 2).

**Table 2.** Summary of litigation with AIA as plaintiff (data from Lex Machina).

Defendants (other parties)	Number of lawyers	Filed/Terminated	Time to termination	Patents asserted	Number of docket entries
Avid Trustees of the Penn (USF Board of Trustees)	12 (four firms) 5 (two firms) 7 (two firms)	2010-11-24/2013-03-19	846 days with no dispositive outcome on patent finding — dismissal	5,455,169 7,538,258 7,807,135	344
Comentis, Inc. Oklahoma Medical Research Foundation	2 (one firm) 4 (two firms)	2009-06-22/2009-11-13	144 days with no dispositive outcome on patent finding—inter district transfer	5,455,169 5,795,963 6,818,448	58
Oklahoma Medical Research Foundation Comentis, Inc. (Walter L. Fast, Ph.D.)	8 (three firms) 4 (three firms)	2009-12-14/2012-12-17	1099 days with no dispositive outcome on patent finding—dismissal	5,455,169 5,795,963 6,818,448	210
Elan Corporation, PLC. Eli Lilly & Co. American Peptide Company, Inc.	11 (two firms) 1 (one firm)	2010-02-02/2012-08-03	931 days with no dispositive outcome on patent finding—dismissal	5,455,169 5,795,963 6,818,448 7,538,258	352
The Jackson Laboratory AnaSpec, Inc. Immuno-Biological Laboratories, Inc.	8 (three firms) 1 (one firm) 3 (two firms)				
Phoenix Pharmaceuticals, Inc. Life Technologies Corporation					
Elan Pharmaceuticals, Inc. (Michael J. Mullan, Ronald E. Sexton, USA Department of Justice)	7 (two firms)				
Myriad Pharmaceuticals, Inc. Mayo Clinic Jacksonville, Inc.	4 (two firms) 4 (two firms)	2003-12-18/2005-06-01	531 days with interdistrict transfer	5,455,169 5,795,963	40
Mayo Foundation for Medical Education and Research	4 (two firms)	2005-06-06/2012-12-27	2761 days with no dispositive outcome on patent finding— procedural consolidation		
Myriad Genetics, Inc.	4 (two firms)				
Myriad Pharmaceuticals, Inc.	7 (four firms)				28
Mayo Foundation for Medical Education and Research	7 (four firms)				
Mayo Clinic Jacksonville, Inc.	7 (four firms)				
Myriad Genetics, Inc.	7 (four firms)				
Pfizer, Inc.	10 (two firms)	2009-06-30/2010-11-12	500 days with likely settlement — stipulated dismissal	5,455,169 5,795,963 6,818,448	111

While protracted and intricate civil litigation without a clear disposition on the merits is common the USA, it is also a hallmark of patent trolls that sue multiple practicing entities simultaneously. It should be noted that the structure of US federal civil litigation is designed to favor private settlement and procedural resolutions over substantive decisions. This structure favors trolls, whose business model is, in part, based on extorting royalty payments that are lower than the costs of defending a patent suit. Avoidance of dispositions on validity of patents likewise benefits trolls, whose patent holdings likely include some of questionable validity. Here, we present a chronological account of litigation that directly or indirectly involved AIA and its patents over the Swedish mutation. The patents expired in October 2012.

### Alzheimer's Institute of America, Inc. v. Mayo Clinic et al.

On December 18, 2003, AIA filed a patent infringement suit against the Mayo Clinic, Jacksonville, Inc.; the Mayo Foundation for Medical Education and Research; Myriad Genetics Inc.; and Myriad Pharmaceuticals Inc. asserting the ‘169 nucleic acids and the ‘963 protein patents.^83^ The suit was over the use of *APPswe*-expressing cell lines that, AIA argued, were not covered under a 1996 license that Mayo obtained from AIA for transgenic mice. According to one article, when the case was moved to Florida,^84^ ‘an arbitrator concluded that Mayo was not allowed to use the *APPswe* cell lines under the licensing agreement with AIA, and that AIA was not entitled to additional money for benefits Mayo received through third-party agreements’.^85^ This conclusion kept the case alive, even though both Mayo and Myriad relied on numerous defenses related to the validity of the patents, with the issues of inventorship and ownership ultimately decided in *AIA v. Avid*, discussed below.^86^

After close to a decade, there was no dispositive outcome on the patent issues at bar in *AIA v Mayo*. However, since Mayo agreed to license the transgenic mice patent carrying the Swedish mutation from AIA,^87^ it is worth digressing from the main tale to discuss another patent infringement suit brought by Elan Pharmaceuticals against the Mayo Foundation, in which the AIA patents played a key evidentiary role. It is also important to note that this case over cell lines contradicts Sexton's contention that AIA did not sue non-profit research organizations.^88^ Sexton claimed that AIA's license to Mayo over transgenic mouse lines facilitated their distribution to the research community and acted as evidence that AIA's intent was not to impact Alzheimer's disease research by the academic sector. However, AIA continued the suit with Mayo over cell lines. To that extent, AIA was, indeed, enforcing its patents against academic researchers at Mayo.

### Sidebar: Elan Pharmaceuticals, Inc. and Athena Neurosciences, Inc. v. Mayo Foundation for Medical Education and Research^89^

In 1996, Irish pharmaceutical company Elan acquired the California-based Athena Neuroscience, valued at ${\$ }$630 million USD.^90^ Athena Neurosciences, Inc., was founded in 1986 and specialized in ‘discovery, development and marketing of products and services to be used primarily by neurologists for the treatment and diagnosis of neurological disorders’.^91^ With the acquisition, Elan gained control over the London mutation patents that Imperial College had licensed to Athena Neuroscience, as well as other Alzheimer's-related patents. The two patents that Elan asserted against the Mayo Foundation in 1999 were US Patent No. 5,612,486 (‘Transgenic Animals Harboring APP Allele Having Swedish Mutation’) with a priority date of October 27, 1993 and US Patent No. 5,850,003 (same title, except ‘Rodents’ substituted for ‘Animals’; same priority date). The patents claimed rodents with the Swedish mutation that produce ATF-betaAPP in detectable quantities in mouse brain homogenate. Inventors on the patents were Lisa McConlogue and Jun Zhao; both were researchers at Athena Neurosciences and then at Elan. No one on the Hardy team was listed as a co-inventor.

Elan claimed that Mayo infringed its patents by making, using, and selling mice that overexpress APP. News coverage of the litigation noted concern within the neuroscience research community about the chilling effect on Alzheimer's research, which at the time had few animal models.^92^ The concern was exacerbated by Elan's subpoena of the laboratory notebooks of key researchers, including some who had made their own transgenic mouse models.^93^ More than 50 academic research groups and a dozen pharmaceutical companies accessed Mayo's transgenic mice, some of which were based on the work by Karen Hsiao's team at the University of Minnesota. That group created Tg2576 mice, which expressed amyloid in the brain.^94^ Mayo licensed Hsiao's mouse as well as AIA's ‘258 patent for a transgenic mouse expressing an APP 670/671 (*APPswe*) mutation in order to be able to distribute the mouse models through Taconic, a commercial mouse breeder and distributor.^95^

As Mayo licensing executives explained:

Mayo has covered the cost of breeding and genotyping Tg2576 mice that are free of specific pathogens… [M]any mice have been distributed to academic researchers. Recipients were asked only to pay a nominal fee, primarily to defray the shipping charge. Despite the lawsuit, we will continue to support Hsiao Ashe in distributing Tg2576 mice to academic researchers.^96^

To further improve accessibility for researchers, Mayo also changed its licensing practices to remove a reach-through provision. That provision had retained rights for Mayo to purchase rights to any intellectual property generated using the Tg2576 mice through a negotiated agreement with mutually acceptable terms.^97^ Elan, on the other hand, closely held its mice for its own pharmaceutical development efforts, which never resulted in an FDA-approved treatment for Alzheimer's disease.^98^

Mayo's motion for summary judgment on the invalidity of the patents was granted by Judge Alsup of the Northern District of California.^99^ The issue was whether the AIA's ‘258 transgenic mouse anticipated the Elan transgenic mouse patents, rendering Elan's patents invalid for lacking the required novelty.^100^ The prior art must expressly or inherently describe all of the elements and limitations of the invention as claimed in the patent being evaluated. However, the prior art need not have been put in practice—Mullan himself never made transgenic mice—but merely must be disclosed fully and clearly enough that someone of ordinary skill in the art *could have* made the invention.

Judge Alsup initially concluded that the ‘258 patent anticipated Elan's patents because it described a variety of methods to make a transgenic mouse, and Elan had, in fact, used one of these. In terms of the distinguishing feature of Elan's patents that the mice had to produce detectable quantities of ATF-betaAPP; he concluded that such mice were simply a subset of the transgenic mice claimed in the ‘258 patent and were covered by its claims. He therefore invalidated the patents Elan had asserted against Mayo. Coverage of the decision exclaimed ‘Neuroscientists worldwide can continue to enjoy access to an important transgenic mouse used for research into Alzheimer's disease’.^101^

However, this optimism was short-lived, as Elan appealed the decision to the US CAFC.^102^ The CAFC disagreed with Judge Alsup, accepting Elan's argument that ‘Mullan does no more than teach broad known “recipes” for gene transfer, and that the Mullan disclosure is simply an invitation to experiment, with no assurance of success’.^103^ The CAFC also observed that ‘[a]lthough Mullan described known procedures for making a transgenic animal, he neither described every element of the claims, nor taught, in terms other than by trial and error and hope, production of a transgenic mouse having detectable ATF-betaAPP in brain homogenate’.^104^ The CAFC therefore concluded that ‘a novel patented product is not “anticipated” if it did not previously exist’—and that the invention claimed by Elan's patents did not previously exist, at least as far as Mullan's ‘258 patent was concerned.^105^ Since Mullan did not make a transgenic mouse, and he did not state which of the methods for making transgenics might work in practice, his patent did not anticipate Elan's.

The 2002 CAFC decision was, however, not the end of the litigation. In 2003, the CAFC issued an *en banc* decision that vacated (replaced) the earlier CAFC decision on anticipation.^106^ The 2003 judgment concluded that Elan's arguments were more appropriately characterized as encompassing enablement. ‘Enablement requires that “the prior art reference must teach one of ordinary skill in the art to make or carry out the claimed invention without undue experimentation”.’^107^ Whether experimentation is undue requires consideration, from the point of view of persons experienced in the field of invention at the time of the filing date of the patent.^108^ The experimentation, therefore, must be more than purely routine,^109^ but it is not necessary for the disclosed invention to have actually been made.

The CAFC (2003), therefore, re-characterized the issue as:

whether [Mullan's] teachings enabled a person of ordinary skill, without undue experimentation, to produce the desired transgenic mouse. This is doubtful considering the reliance in the ‘258 patent on conditional statements, for example, “how vectors can be constructed”, the transgene “can be injected”, and other similar statements… Mullan does not suggest which, if any, of the methods and vectors he cites might reasonably be predicated to succeed in producing a mouse operatively harboring the Swedish mutation.^110^

Since Judge Alsup had not considered enablement arguments in his initial judgment, the CAFC remanded the case back to the District Court for reconsideration, with instructions to assess whether the enablement criterion was met. However, the District Court never had the opportunity to rule on enablement. On November 12, 2004, Elan and Mayo settled the legal dispute that allowed both to use a range of research tools, including the Tg2576 mouse.^111^

If reconsideration based on enablement had, in fact, occurred, it would likely have been to the detriment of Mayo. The CAFC opinion in 2003 recognized strong arguments that the ‘258 patent did not enable Elan's patents (see discussion below), in which case Elan's patents would have been valid and enforceable. This same argument, however, casts doubt on the validity of the ‘258 patent, since patents require sufficient disclosure specifications to enable a person of ordinary skill in the art to make the invention claimed. While it was never fully litigated, the CAFC's analysis clearly suggests the ‘258 patent was potentially invalid due to incomplete enablement.^112^

There are two further points that arise from this litigation. First, the CAFC (2002) posed a pertinent policy question. In response to the dissent, Judge Newman stated that:

although our colleague postulates “serious and unfortunate consequences in the future” if the Elan mouse is deemed patentable, others may believe that without the possibility of a patent on a new transgenic mouse, the hypothetical mouse envisioned by Mullan might well remain no more than a hypothesis.

It is clear from the record, however, that Judge Newman's concern was, while understandable, demonstrably misplaced. Patent incentives were not the driving force for researchers to create most of the transgenic mouse models for Alzheimer's research. Indeed, Hsiao's mice were created, and they were freely distributed to the community for further research. The Tg2576 mouse had become one of the most widely used transgenic models for Alzheimer's, despite uncertainties about user rights.^113^ Many groups around the world were attempting to generate mouse models. At present, the International Mouse Strain Resource^114^ indicates 235 strains of APP mouse models available globally as live mice, sperm, or embryos. In the 1990s, lack of transgenic mouse models was due to technical complexities, not lack of commercial or patent incentives.

Second, when Elan asserted its patents against the Mayo Foundation in 1999, He enjoyed record profits of more than ${\$ }$1 billion USD annually. It is clear, therefore, that the aim of the litigation was primarily to safeguard the value of Elan's in-house Alzheimer's research by limiting the supply of research models to rival companies.^115^ While this business decision was based on patent rights, and might be contrary to the norms of sharing within the research community, it fits squarely within the patent system's economic justifications of exclusivity and competitive advantage for a company that was practicing the patent. Mayo was, via Taconic, distributing its licensed mouse lines to industry for up to ${\$ }$850,000 per breeding pair and was therefore a target for suit, with resulting implications for academic researchers in access to research tools if Mayo were forced to cease distribution.^116^

From a societal/public policy perspective, the Elan case also presents the cautionary tale of ‘putting all eggs in one basket’ through the monopolization of key research tools for pre-clinical research. In 1999, Elan researcher Dale Schenk and his team raised the possibility, using Elan's transgenic mouse model, that immunization with amyloid-β might be effective in preventing and treating Alzheimer's disease.^117^ Elan commenced phase I clinical trials in 104 participants from the US and UK, testing the safety of various doses of synthetic A-beta 42. Preliminary results were encouraging and news coverage hailed the approach as revolutionary with the potential to change the therapeutic landscape for Alzheimer's disease.^118^ Investment analysts were bullish: after the completion of the successful phase I trial, shares of Elan were trading at over ${\$ }$60 USD.^119^ With a new partner, Wyeth-Ayerst, Elan began enrolling 352 participants with mild to moderate Alzheimer's disease in a phase IIa multicenter clinical trial in Europe and the US Then, on January 17, 2002, the trial was suspended because four French participants developed encephalitis. By March 1, a total of 15 participants had developed encephalitis and the trial was permanently halted with concomitant reductions in value of Elan stocks.^120^ Thus, Elan's approach had failed like most therapeutics in phase II.^121^

The Elan case, therefore, represents a clear case where a broad and diverse research base can be preferable, as a matter of public health policy, for addressing a disease as devastating and complex as Alzheimer's disease. It is important to pursue a wide range of therapeutic approaches. Such research must be supported by easy access to research tools, such as transgenic mice. This example shows that aggressive enforcement of patents over research tools stifled research by increasing its costs while there was little evidence that patents created the key incentives to develop such tools.^122^ To the extent that patent incentives were important to Elan, the patents were used to restrict competing research by blocking access to transgenic mouse models of Alzheimer's disease despite the questionable scope of Elan's patents over those mouse models. Judge Newman's concern for losing patent incentives to create new transgenics was backwards in this particular case. The prospect of patents was not a crucial incentive for transgenic model development, but instead patents were actually used to block access to transgenic mice that had already been developed.

### The JAX Litigation: Alzheimer's Institute of America, Inc. v. Elan Corp. et al.

In this litigation, AIA asserted four patents against nine defendants (Table 2). Several patents held by AIA ‘describe the use of assays, cell lines, and animal models including the APP Swedish mutation’.^123^ Some of the defendants sold *APPswe*-containing peptide sequences and other reagents. However, many of the defendants settled, and litigation against the remaining, Elan and Eli Lilly, was subject to the disposition in *AIA v. Avid RadioPharmaceuticals et al.*, discussed below.

We now turn to the litigation against the Jackson Laboratory (JAX). AIA asserted only its ‘258 transgenic mouse patent against JAX, alleging that 22 of the mouse strains in the JAX repository infringed its ‘258 patent.^124^ In 2011, AIA attempted to amend its claim also to enforce the '160 Patent which it ‘inadvertently failed to assert’.^125^ US Magistrate Judge Laporte denied the amendment motion largely because the delay of adding it would derail the scheduled steps in the litigation, in particular, a Markman hearing on claims interpretation. The delay would unacceptably increase litigation costs.

Magistrate Judge Laporte described JAX as ‘a non-profit academic institute that uses and sells transgenic mice as a research tool, including for use in Alzheimer's research’.^126^ JAX supported community academic and publication standards as well as being National Institutes of Health (NIH) contractor to make mice used in research available to researchers.^127^ Funding a public repository such as JAX lessened the burden on individual laboratories and enabled wide use of transgenic Alzheimer's mouse models.^128^ Judge Laporte agreed with JAX's opposition statement that:

the majority of the mice available from Jackson's Alzheimer's repository sell in low volumes, and Jackson loses money on sales of mice from low volume strains. Jackson is therefore only able to distribute the accused Alzheimer's mice through the generosity of private philanthropy and federal government (NIH) grants.^129^

Further, JAX ‘provides the accused mice only to non-profit researchers, and loses money doing so’.^130^ In addition, ‘patent litigation relating to its mice is chilling progress towards an Alzheimer's cure’.^131^

Concern about the chilling effect of patent litigation on research was a theme in media coverage of the case.^132^ Quoted from an interview, David Einhorn, JAX in-house counsel, reiterated a continued commitment to distributing the mice but worried that ‘moving forward, people may be less willing to donate mice to our repository, or even use [Alzheimer's disease] mice because of the fear of being drawn into litigation’.^133^ He explained that institutions had been reluctant to deposit mice into the JAX Alzheimer's disease repository because, even though scientists might be willing, their institutions:

have felt hamstrung, or at least confused, by the complex patent landscape surrounding research tools involving [the Swedish mutation], *APPswe*. As such, patents or pending patent litigation have impeded [Alzheimer's disease] research for much of the past decade by making it hard to obtain key [Alzheimer's disease] mouse models *or make new ones*.^134^

In at least one instance, Einhorn was aware of an institution that AIA had threatened withlitigation demanding a substantial license fee. Despite the threats, the institution sent the mice to JAX, although some years later.^135^

Sexton responded to a 2010 article by Landhuis and Strobel and an equally critical 2011 article by Erika Check Hayden in *Nature*. He contended that AIA did not threaten academic researchers with litigation and indeed supported such research.^136^ He claimed that ‘Jackson is selling the mice and making quite a lot of money in the process. Furthermore, the mice Jackson is selling are, in many instances, being used for commercial purposes—not academic purposes’.^137^ As an example, he cited the fact that JAX provided the mice to University of Pennsylvania (Penn) researchers, who then used the mice to develop commercial imaging agents. Penn then spun out a company, Avid RadioPharmaceuticals (Avid), which was then acquired by Eli Lilly for up to ${\$ }$800 million USD.^138^ US patent law's explicit statutory framework for commercializing university research makes Sexton's argument particularly relevant. His placement of the line between academic and commercial research^139^ moved commercial research into academic institutions, and indeed highlights important questions about the proper role of commercial motivations in academic research that is also publicly funded.^140^

However, JAX's greatest concern during the litigation was the AIA demand that JAX reveal the names of researchers to whom it had distributed mice.^141^ JAX refused to settle because that would mean handing over the names of researchers who would then face potential lawsuits.^142^ Geneticist Mike Sasner of JAX stated in an interview, ‘We knew [AIA] was prepared to sue those researchers if any developments came as a result of their using the mice [as they had done to the University of Pennsylvania]. At that point, we realized we needed somebody to intervene, and that the government had an interest in doing this’.^143^

JAX was left with the impression, from these demands for scientists’ names, that AIA intended to assert its patents against the institutions of Alzheimer's disease researchers who had either developed novel *APPswe* lines, or who had used lines distributed *b*y JAX for research. For this reason, in a letter dated December 15, 2010, JAX requested support from the National Center for Research Resources (NCRR).^144^ JAX cited funding support from NCRR and the fact that the AIA patent over the transgenic mice, as the result of a continuation application in the US Patent and Trademark Office (USPTO), was granted only in 2009, 17 years after the first patent over nucleotide sequences was filed in 1992. AIA sought not only damages, but also the discontinuation of JAX's distribution of the 22 mouse models for Alzheimer's research. If this had succeeded, the foremost center for distributing Alzheimer's disease animal models for research would have been hamstrung. Citing other AIA litigation, JAX also emphasized the fear of litigation that had spread throughout the academic community, hampering the deposit, and distribution of mouse lines.

In practical terms, JAX requested that NCCR confirm that ‘the federal government supports and stands behind Jackson's distribution of Alzheimer mouse strains’.^145^ JAX asked NIH and its Office of General Counsel to request that the Department of Justice intervene in the litigation as an interested party, given the investment of federal funds in Alzheimer's research. But more importantly, JAX requested a letter of *Authorization and Consent*.^146^ The effect of *Authorization and Consent* is to relieve a government contractor—in this case, JAX—from patent infringement liability, effectively shielding JAX from infringement lawsuits and substituting the federal government as defendant in the event that a patentee still wished to pursue claims of infringement.^147^ As in this case, *Authorization and Consent* may be granted post hocduring infringement litigation.^148^

On June 17, 2011, NIH Director Francis Collins agreed to provide JAX with *Authorization and Consent* to use and manufacture any US patented invention to access, develop, and distribute transgenic mouse models of Alzheimer's disease under specific grants to JAX from NCCR, the National Institute on Aging, and NIH. The letter covered the 22 mouse strains that were the subject of the litigation and that had been brought to JAX under the support of a grant from NCRR. On August 10, 2011, litigation against JAX was dismissed without prejudice, with each party bearing its own attorney fees and costs.^149^ In the agreement between the parties, dated August 9, 2011, AIA agreed to a covenant not to sue JAX for any past or future infringement. JAX denied all AIA infringement allegations. NIH Director Collins affirmed that ‘provision of these important research tools to scientific investigators… is critical to the advancement of our understanding of Alzheimer's disease and to the development of new diagnostics and treatments for this devastating disease’.^150^

The dismissal of the suit against JAX was widely hailed as positive. Einhorn stated:

[W]e hope and trust that the dismissal will encourage researchers and institutions who have been inhibited by the fear of being sued to use mouse models in their Alzheimer's research… As well, we trust that the intervention of the NIH on behalf of Jackson will encourage researchers who develop new mouse models, both of Alzheimer's and other major diseases, to provide them to the Jackson mouse repository so they can be shared with the rest of the research community.^151^

David Holtzman of Washington University School of Medicine in St Louis, Missouri, concurred: The mice distributed by the JAX have made a major contribution in allowing investigators all over the world to make fundamental basic and disease-related insights into Alzheimer's disease. This type of effort should only get to be a bigger, not smaller, enterprise until we have solved this disease.^152^

### Alzheimer's Institute of America, Inc. v. Avid RadioPharmaceuticals et al.

AIA asserted two patents against the Penn and its spin-off company Avid, the ‘169 nucleic acid and the ‘258 transgenic mouse patents (Table 2).^153^ AIA alleged that researchers at Penn used Tg2576 mice to test imaging agents. Penn then patented the imaging technology and licensed it to Avid. The mice were sourced from JAX. The Penn patents were evidence of the use of AIA's patented technologies. AIA sued Avid on the basis of a publication by Avid's founder and CEO, Dr Skovronsky, that used a mouse model to detect amyloid plaques.^154^ In 2010, Avid was acquired by Eli Lilly and Company (Lilly) for ${\$ }$800 million USD.^155^ Moreover, in a closing of the loop, Lilly had entered into agreements with Athena Neurosciences, which had an exclusive license with Imperial College to both research and patents from Hardy's team on the *APP* gene. The defendants therefore argued that Imperial College had an ownership interest in the Swedish mutation, an interest which, if proven, would have given Avid/Lilly the rights to use the invention.^156^ Other defenses to invalidate the patents included anticipation, obviousness, and failure to satisfy the written description, enablement, and/or best mode requirements.^157^ The argument that prevailed at trial, however, was failure to name the true and correct inventors with deceptive intent.

A threshold issue in all litigation is whether the plaintiff, in this case AIA, has standing to bring the action. For patent infringement actions, if the plaintiff does not own the patent, it has no standing. Among the foundational arguments before Judge Savage in this litigation, therefore, was on motions for summary judgment on whether AIA owned the patents. Since summary judgment cannot be granted if there are disputed material facts^158^ and Judge Savage found that factual disputes did remain, his 2011 Memorandum Opinion denied summary judgment, and the issues on standing were heard by a jury at trial.^159^ The jury found against AIA, but there are no written reasons, just the jury's verdict.^160^ AIA then filed a post-trial motion seeking to set aside the jury's verdict and for Judge Savage to grant either judgment in AIA's favor as a matter of law or else a new trial. The 2013 Memorandum Opinion by Judge Savage addressed this post-trial motion and added clarity to the jury verdict.^161^ Here, we summarize both the 2011 and 2013 Memorandum Opinions.

The two arguments against standing brought by the defendants were that (1) Mullan was not the sole inventor on the patents and therefore the patents were invalid and (2) even if Mullan were the sole inventor, the ownership of the patents vested in USF by virtue of Mullan's employment at USF and the operation of Florida law. We address inventorship and ownership in turn.

#### Inventorship

The inventorship issue hinged on whether others should have been named as co-inventors on the AIA patent.^162^ Inventors are individuals who contribute to the conception of the patentable invention,^163^ and joint inventorship is governed by the Patent statute.^164^ Each inventor must be named in a patent application.

The initial statements of defense stated that members of the Swedish team should have been named as co-inventors.^165^ In AIA's suit against Mayo and Myriad (Table 2), Lannfelt, at least had been deposed on the issue of inventorship.^166^ Lannfelt and Winblad had allegedly claimed co-inventorship at the time the patents were still pending but had failed to disclose the dispute to the USPTO.^167^ In an interview with AlzForum, Lannfelt stated that following his visit to Hardy at Imperial College, the sequencing of the Swedish samples was split between Mullan (exon 16) and Lannfelt (exon 17). The mutation was located on exon 16, and the ensuing publication had Mullan as first author and Lannfelt as last. As he recalled: ‘It was a good collaboration and I have never regretted it. Then [Mullan] told me he was going to take out a patent and said you can only have one name on it. This was untrue. But in 1992, I knew nothing about patenting mutations’.^168^ In terms of the discovery of the *APP* gene, Lannfelt stated, ‘I would have found the mutation without Mullan, though later. He would never have found it without us’. Mullan, on the other hand, claimed that Lannfelt and Winblad did not deserve recognition on the patents because ‘they only provided DNA, and made no intellectual contribution’.^169^

At trial, the issue of inventorship focused on whether team lead, Hardy, should have been named as co-inventor. As an inventor, Mullan made a legal ‘oath and declaration’ that he believed himself to be the original and first inventor. Since the issuance of the patent created a presumption that Mullan was the sole inventor, the onus was on the defendants to prove otherwise by ‘clear and convincing evidence’, a standard only slightly more relaxed than the stringent ‘proof beyond a reasonable doubt’ necessary in criminal law. Given the degree of collaboration between Hardy and Mullan, it was reasonable for the jury to conclude that the invention was ‘the product of a collaboration between two or more persons working together to solve the problem addressed’.^170^ That collaboration need not have been coincidental in time or in location. The joint inventor need only have made ‘a significant contribution on the road to and not necessarily at reaching conception’.^171^ The evidence of Hardy's contributions was supported not only by Hardy, but also by contemporaneous documents, such as laboratory notebooks and correspondence, and witnesses who had been part of the Imperial College research team. Both Hardy and another team member testified that Hardy and Mullan held off sequencing until they arrived in Florida and this ‘could have been considered by the jury as both attempting to deceive Imperial College and USF.… the jury could have concluded that omitting Hardy as a co-inventor on the patent application was part of the ploy to defraud Imperial College and Athena’.^172^ The evidence showed that Hardy was involved in, and directing, the process to discover the mutation, leading to the conclusion that he substantially contributed to the invention.

#### Ownership and waiver

The issue of initial ownership of the patents is central because, if Mullan never owned the patents, then he had no ownership interests to assign to AIA, meaning that AIA also could not own and enforce the patents. Mullan conceived of the inventions when he was employed at USF, and Hardy was employed by both Imperial College and USF. The issue of ownership, therefore, was answered in the first instance by Florida law, which vests ownership of any inventions developed or discovered by an employee in the course of employment with USF.^173^ Employees of USF are under a duty of disclosure to the University,^174^ but USF reserves the right to relinquish its ownership interest. Because Mullan was an employee of USF and pursuing Alzheimer's disease research, which he was hired to do, ownership of the patents automatically and immediately vested in USF by operation of Florida law. In such a case ‘the inventor has no property rights to assign, and any purported assignee lacks standing to sue for patent infringement’.^175^

The real issue, therefore, was whether Florida knowingly waived its rights to the invention. Under the law, the inventor had the duty to disclose the invention at which time USF could elect to transfer its rights to the employee. However, to be effective, such an assignment or release of rights ‘must contain a provision that the invention remains available royalty-free to the State of Florida for government purposes’.^176^ The ‘unequivocal’ evidence for waiver relied on by AIA was the letter signed by the Vice President of Research on May 4, 1992, that stated, ‘[A]ll ownership of rights in any work carried out by [Hardy and Mullan] and inventions made by them before August 15, 1992 belong exclusively to Hardy and Mullan’.^177^ However, there was evidence that would have led a reasonable jury to conclude that: ‘Newcome did not intend to waive USF's rights to the Swedish mutation invention’.^178^ Evidence showed USF administrators were aware of Hardy and Mullan's intellectual property disputes with Imperial College and wanted to draw a line between work done in the UK on the London mutation and work done at USF. USF administrators believed that Mullan was in the process of setting up Hardy's laboratory and, therefore, could not have started new work at USF. They were unaware of his off-campus sequencing of the Swedish mutation. The cut-off date in the letter reflected time for Hardy to complete work at Imperial College and the start of the new semester at USF.^179^ No further actions or correspondence showed that Newkome was ever aware that the Swedish mutation had been discovered while Mullan was employed by USF. In conclusion, ‘[t]he jury's determination that USF did not knowingly and intentionally waive its rights because Newkome lacked sufficient knowledge to waive a known right was amply supported by the evidence’.^180^

#### Appeal to the CAFC

Following the jury trial and the 2013 Memorandum Opinion denying AIA's motion for a new trial, Sexton stated:

for almost 20 years, AIA has been and continues to be the record owner of title to the Swedish mutation patents… Michael Mullan's inventive contribution to the discovery of the Swedish mutation and the resulting invention was not disputed in the litigation and remains unchallenged. Per its policy, AIA will not comment further on pending litigation, other than to state that it remains ongoing in the trial court and will likely continue in the US Court of Appeals for the Federal Circuit.^181^

However, others commented that success was unlikely because both parts of the jury verdict, inventorship and waiver, would need to be reversed.^182^ That prediction proved correct. Without giving reasons, the three-judge CAFC panel (Judges Newman, Plager, and Moore) affirmed the 2013 Memorandum Decision of Judge Savage on May 16, 2014.^183^

#### Disposition on other AIA litigation

Following the final disposition in *AIA* v. *Avid* (2014), all further ongoing litigation (Table 2) was discontinued.^184^ The only outstanding legal actions involve disputes over costs, all of which were awarded against AIA. However, some defendants, such as the Mayo Foundation, agreed to licensing terms on some inventions and many of the co-defendants in the Elan litigation had settled.^185^ As noted by Landhuis (2012): ‘One question raised by the recent court finding is that defendants in previous suits brought by AIA who have chosen to settle may now consider suing AIA on the argument that AIA never had standing to bring infringement claims on a patent it did not own’.^186^

## LEGAL ANALYSIS OF PATENT VALIDITY

The AIA's litigation failed, but it is worth briefly reviewing substantial legal barriers that would have stood in the way of patent enforcement even if AIA had been deemed the rightful owner of patent rights. The claims in the *APPswe* patents were, as noted, highly vulnerable on grounds of enablement for the transgenic mouse claims. But what about the claims on the methods and the DNA molecules claimed in the patents?

Both method and composition of matter (DNA molecule) claims would also have been quite vulnerable to challenge, because the claimed invention was based on finding *APPswe* mutations in DNA derived from human samples. Patent claims covering those mutations would therefore contravene Justice Clarence Thomas's unanimous Supreme Court decision of June 13, 2013, which states: ‘a naturally occurring DNA segment is a product of nature and not patent eligible merely because it has been isolated, but that cDNA is patent eligible because it is not naturally occurring’.^187^ Claims to *APPswe* mutations themselves, or isolated DNA containing them, would thus be invalid, although DNA constructs engineered to create cell lines and transgenics would be eligible to patent. Such claims to engineered DNA would have to be new, useful, non-obvious, fully enabled and adequately described. Given the state of Mullan's science at the time of patent application, which largely amounted to having discovered the *APPswe* mutations, these would be high hurdles to clear. As noted, Mullan never created cell lines or transgenic models. The method claims in the AIA patents might also have been judged invalid, based on another unanimous decision of the US Supreme Court in *Mayo v Prometheus*.^188^ That 2012 case Supreme Court decision invalidated claims on diagnostic methods that claim a ‘law of nature’ with insufficient additional invention. The likelihood that the Mullan method and DNA molecule claims would be judged invalid and which are further corroborated by the December 2014 decision of the CAFC in *University of Utah v Ambry Genetics* (also known as *In Re: BRCA1 and BRCA2-based Hereditary Cancer Test Patent Litigation*).^189^

While the validity of the patent claims granted to Mullan is moot because the patents were invalidated on grounds of standing and inventorship, the question of whether patent rights on transgenic animal models were granted appropriately to Mullan nevertheless bears scrutiny because of the centrality of the transgenic mouse claims in all the litigation that followed from having discovered the *APPswe* mutations. Given the CAFC's analysis of enablement in the *Elan* litigation, it seems likely the USPTO granted patent claims for transgenic mice with *APPswe* mutations prematurely and to the wrong inventor.^190^ The ‘258 patent claimed an *APPswe* transgenic mouse (Table 1), which expresses the human *APP* or a fragment thereof with the Swedish mutation.^191^ The specifications, under the heading ‘Production of Transgenic Animals with Mutant APP Allele’, describe methods for the generation of the nucleotide sequence construct for insertion into the mouse, followed by very short descriptions for preparing the DNA for injection, microinjection, and one sentence on identifying a transgenic mouse. The patent references only a generic laboratory technique manual that arose as a result of courses at Cold Spring Harbor.^192^

Few researchers could make transgenic mice in 1992. Core facilities for mouse model work were in their infancy. The first publication on a mouse containing foreign DNA, generally inserted via pronuclear injection of mouse zygotes, was first published in 1980^193^ with first expression of a transgene in 1981.^194^ The OncoMouse, which in the 1980's was genetically modified with a mouse (not a human) gene to exhibit a predisposition toward cancer, was developed and patented by researchers at Harvard University.^195^ But making such transgenic mice was technically complex and required highly skilled personnel. With the success of the OncoMouse, there was hope that models for other diseases would rapidly follow. The skills to make transgenic mice spread slowly from key research centers, with the first publication of relevant protocols being the Cold Spring Harbor laboratory manual, which began as a ‘help’ manual for an annual course at the laboratory.^196^ Evidence for the novelty and complexity of the creation of transgenics in the early 1990s is the fact that researchers could announce new transgenic mouse models in high impact journals, which is no longer the case.^197^

This certainly suggests that a patent that claimed transgenic mice in 1992 would likely have been non-obvious over the state of the prior art. The inventive gap would have been large between the prior art and the claimed transgenic mice, counseling in favor of separate patentability of transgenic animals beyond mere discovery of a mutation sequence. The broader the patent claim, the more detailed the patent disclosure must be in order to adequately fill that inventive gap with enabling instruction. In other words, if an invention is clearly non-obvious because ordinary skill in the art is low, then the inventor tends to face a greater disclosure burden to more fully raise that level of skill. Conversely, if a sparse disclosure is enough because ordinary skill in the art is high enough to fill the gaps, then that higher level of skill also tends to undercut non-obviousness. By design, the requirements of non-obviousness and enablement are in some tension with each other so that the public may enjoy the full benefit of the patent bargain by receiving broad instruction in exchange for granting broad patent rights. The problem with the AIA patents was inadequate enablement, for the state of the art grew slowly despite the patent disclosure.

It was not until February 1995 that researchers at Athena Neurosciences, Exemplar Corporation, Lilly Research Laboratories, the Scripps Research Institute, the University of California at San Diego, and the Laboratory of Clinical Science of the National Institute of Mental Health published in *Nature* the first transgenic mouse model that overexpressed the V717F β-amyloid precursor protein.^198^ According to Penn pathologist John Trojanowski, this was the ‘first mouse “that really shook the world”’.^199^ Indeed, Tanzi, describes it as a ‘sensational discovery’, and in an accompanying article, Karen Duff and Hardy stated that ‘creating such a model [as the Athena mouse] has been no easy task’. This paper goes on to pose questions about ‘the production of these remarkable animals’.^200^ These relate to the technical difficulties and uncertainties in producing the models:

what element of the construct was crucial to its success (the promoter, the use of introns or the use of mutations?) Will mice containing one or more pathogenic mutations carried on full genomic constructs… be even better?… The answers to many of these questions [including about the pathophysiology of Alzheimer's disease] will now come quickly, especially if these mice are made available to the general research community. Clearly expanding and distributing colonies of the mutants should be a priority.^201^

The latter quote from Hardy is ironic given his own attempts to patent transgenic mouse models of the London mutation and license these to Athena while still at Imperial College.

According to Tanzi:

the Athena mouse model originally had been engineered by scientists at the biotech company TSI, Inc. Apparently, TSI didn't realize how earthshaking its mouse line was, for Exemplar—a division of TSI—put the model up for sale. Athena, upon close scrutiny of the mouse, saw that it was both greatly overexpressing A-beta and producing an Alzheimer's like pathology and immediately bought out Exemplar.

The model, in its development through the summer of 1996 had ‘fifty-some Athena researchers perfecting it’.^202^ Yet, Athena, as discussed above, was reluctant to share the model that was so central to its drug discovery efforts.

A mouse model of the Swedish mutation did not eventuate until October 1996, more than four years after the *APPswe* mutation was published. The transgenic mouse incorporating *APPswe* was developed by Karen Hsiao at the University of Minnesota.^203^ This was the model distributed by Mayo under a licensing agreement with AIA. However, most importantly, a transgenic mouse with a single mutation was still not a useful model for Alzheimer's disease and drug testing, because it did not demonstrate all of the pathophysiology of the disease.^204^ The more valuable transgenic animals, which became the industry standard, had multiple mutations to better model brain plaques, including adding mutations in the presenilin genes.^205^ Nevertheless, with all the resources of academia and the biotechnology and pharmaceutical sectors combined, it took four years from Mullan's claims for other researchers to make an *APPswe* transgenic mouse.

In conclusion, it is clear that in 1992, the creation of a transgenic mouse, by a sole inventor who was a clinician/geneticist with no background in transgenic or mouse research, was highly speculative at best, and granting patents rights on transgenics based on DNA sequence of the mutation was a mistaken evaluation of the state of the art by the USPTO. Three to four years of intensive work by large teams to produce an *APPswe* transgenic mouse is a solid working definition of ‘undue experimentation’, the criterion for invalidity of a patent based on enablement. The USPTO lapse is particularly concerning given the 17-year examination history of the AIA patents at the USPTO—the transgenic mouse patent issued only in 2009 and to the wrong inventor. The continuation problem compounds the fact that AIA did not seem to enable the full scope of what was claimed in its broad claims over transgenic mice. It represents an example of how inadequate enablement can impede downstream research, two decades after the law changed to limit continuation abuse. The case thus also became an instance of ‘submarining’, in which a prior patent is enforced a subsequent inventor who actually reduces the invention to practice. The situation arose here because the priority date was in 1992. All applications filed as of June 8, 1995, and later can gain in strategic issuance delay only what they give up in post-issuance patent term (because the expiration date is 20 years from the priority date of application rather than 17 years from the patent's issuance date).^206^ In other words, the *APPswe* transgenic animal patent was an instance of a systemic problem that was recognized as such and addressed through legislation.

This case instantiates the threat of patent trolls emerging with invalid claims that have been improvidently granted by the USPTO. Examiners spend only limited time with each application and patent offices expend limited resources on initial reviews; both cause problems in rapidly evolving fields.^207^ Commentators have noted the substantial error in examination at patent offices,^208^ and the issue has been raised as compounding the impact of NPEs, such as AIA.^209^ A recent report on *Patent Assertion and US Innovation* points to the need for clearer patents with a high standard of novelty and non-obviousness.^210^ Faster and less expensive mechanism to challenge the validity of patents, such as the new process for post-grant review implemented by the *America Invents Act*, are a promising step to enable rapid review of questionable and overly broad claims that impact research.^211^

## POLICY IMPLICATIONS-ABERRANT FACTS OR TIP OF THE ICEBERG?

This case has implications for policy. One key question is about the value of patents in the research ecosystem; another is about the role of NPEs or patent assertion entities, such as AIA. In 2012, NPEs filed 62 per cent of IP infringement suits in the USA, a tripling since 2010.^212^ Biotechnology in general, and biomedical research in particular, are vulnerable to increasing litigation from NPEs in that there is uncertainty in both the scope of claims and potentially infringing activities.^213^ Assertions of patent infringement are costly to counter; defendants bear the cost of investigation to furnish evidence that their activities have not infringed the patents. In addition, patent litigation ranges from ${\$ }$1–6 million (USD), or even more, depending on the complexity of the case. In light of costs and uncertainty, many defendants settle (as was the case for most defendants here). Settlement amounts:

are affected more by the parties’ relative opportunity costs of going to trial and attitudes towards risk factors that favor [NPEs], whose legal fees are low (since they do not have to provide much evidence to assert there has been patent infringement), and who do not have to pay the fixed costs of a manufacturing operation. Therefore, [NPEs] have an incentive to drag out litigation to increase pressure on defendants to settle the case.^214^

AIA is an early example of litigation brought by an NPE in biotechnology against researchers.^215^ NPEs operate from a position of strength. Since they have no products in the market to threaten, and no research and development operations, they cannot be counter-sued for infringement. NPEs rely on it being more cost-effective to settle or pay a license fee than to engage in litigation. Given the risk aversion of most research institutions, this may make such institutions more vulnerable to NPEs, such as AIA, that are ready to sue non-profit entities.

While AIA lost its patent rights for the idiosyncratic reasons of inventorship and improper assignment, the case illustrates the defenses that may be raised against NPEs, including the many avenues to invalidate patents. Recent jurisprudence on patentable subject matter and supporting USPTO guidelines, stricter standards on utility and new mechanisms to challenge the validity of claims may all favor defendants against overly broad or questionable claims asserted by NPEs. However, other policy options to employ against NPE tactics specifically against research may also be needed.

This case illustrates the use of *Authorization and Consent* as one such option that will be available to entities that qualify as government contractors. While remaining a practical option in some circumstances, the policy solution with the greatest impact is likely a statutory research exemption or specific legislation targeted at controlling the excesses of NPEs. Unfortunately, legislative reforms are difficult to achieve, as illustrated by the protracted debates over patent reforms, including the recent *America Invents Act*.^216^ History suggests that such reforms represent a compromise in which no one stakeholder is fully satisfied. Other policy responses, therefore, should be examined, including judicial intervention, judicial rules for litigation, and policies and guidelines of agencies and institutions that fund and oversee research.

### Legislative reform

In 2013, 14 bills were introduced to the US Congress to address issues related to the actions of NPEs, some of which exempted life sciences companies.^217^ However, none has made it through the US Senate, having been opposed by universities, invention promotion groups, the patent bar and pharmaceutical companies.^218^ These groups found several provisions problematic in the most comprehensive of these bills, the *Innovation Act*^219^. The first was fee shifting, which would make the losing party pay the attorney fees of the prevailing side, a rarity in the US This is in contrast to other countries, such as the UK and Canada where fee shifting is the norm. The *Innovation Act* would punish trolls by shifting costs of abusive litigation tactics to the NPE, if it lost in court. These fees can be substantial in patent litigation. However, the US bar argued such a policy would slide down a slippery slope, that fee shifting would not be limited to patent infringement litigation. A second was joinder, that is, the joining of two or more parties as co-plaintiffs or co-defendants in a lawsuit where they share similar rights or liabilities. Opponents were also concerned about heightened pleading and transparency requirements, which would require all patent owners to detail their infringement claims and accompanying changes to the discovery process in providing documentation to support their claims. In addition, discovery rules would be tightened to discourage the NPE tactic of irrelevant discovery requests, which drives up attorney fees to encourage settlement. The one remaining legislative reform, introduced in July 2014, is the *TROL Act*^220^ which specifically targets patent holders that send opaque and misleading demand letters, making the sending of a demand letter in bad faith punishable as an ‘unfair or deceptive act’ under the *Fair Trade Commission Act*^221^. However, this act only targets a small subset of the problems with NPEs and trolling behavior^222^ and would not, for example, have been effective in the case of AIA, which did not send out large numbers of deceptive demand letters.

Some of these failed provisions in specific legislation, however, have also been the target of judicial rules. Some rules discourage NPEs by targeting specific tactics and mitigate the need for specific legislation targeting problematic troll behaviors. NPEs commonly assert multiple patents against multiple parties, all of whom bear the burden of producing evidence to defend against claims of infringement. NPEs also threaten injunctions, which may damage the operations of innovative firms. Recent SCOTUS decisions on enhanced standards for injunctions in patent disputes and fee shifting,^223^ in addition to *America Invents Act* reforms limiting the number of defendants that can be sued in a patent infringement suit,^224^ all contribute to curbing the litigation excesses of NPEs. With respect to enhanced standards for pleadings, to increase the investigational costs for plaintiff NPEs, on 22 September 2014, the Judicial Conference of the US approved the elimination of Federal Rule 84. The elimination means the end of bare bones pleading forms set up in 1934. The Judicial Conference recommended that SCOTUS approves the change to bring patent pleading in line with recent jurisprudence from that court.^225^ However, the CAFC has resisted heightened pleading in patent lawsuits.^226^

Further legislative reforms were brought in by the *America Invents Act*, which may be helpful in some circumstances, especially in challenging overly broad and questionable claims through the new process for post-grant review.^227^ This mechanism provides a faster and less expensive administrative mechanism than litigation to challenge the validity of patents. Such a mechanism might have been helpful in challenging AIA and has been available in Europe for some time. The European patent opposition system provides third parties with a post-grant opportunity to challenge the validity of European patents without litigation.^228^ Challenges may be brought before the European Patent Office because the patent matter is not an invention, its subject matter is excluded under European patent law, or claims fail one of the three patentability criteria: novelty, inventive step, and industrial application.^229^

In the case of NPEs bringing suits against publicly funded researchers and their institutions, the most comprehensive solution would be a statutory research exemption. The USA has no research exemption or compulsory licensing authority, although the US Government does have use rights subject to ‘reasonable compensation’ to a patent-holder. *Madey v Duke University* made clear the very limited research exemption under US case law^230^—there are fewer constraints on exclusive patent rights reaching into research in the US. Indeed, the precedent set by *Madey* suggests that universities and other research institutions in the USA are not exempt from infringement liability just because they are doing research, and this underscores the desirability of a research exemption. However, recent debates over a research exemption in the *America Invents Act* further illustrate complexities of legislative reforms. Early drafts of the US patent reform bill included a research exemption, but it did not become law. One problem was in the definition—it is difficult to draw clear distinctions between non-commercial research covered by a research exemption, research that is translational, and research with commercial intent. However, Australia has recently clarified its research exemption in its ‘*Raising the Bar’ Act* of 2012.^231^ In line with some European jurisdictions, Australia allows research *on* and invention rather than research *with* the invention, thereby protecting the commercial interests of research reagent companies.

A further mechanism might be a compulsory licensing authority. Such authority was contemplated but rejected in the 1952 revisions to the US Patent Act,^232^ to the chagrin of some legal scholars, who presciently predicted its absence would cause future problems.^233^ The Australian Government also has authority to invoke compulsory licenses, and ‘Crown use’ provisions (for government purposes) that provide escape valves when rights holders thwart public interest.^234^ Those authorities are the subject of a recent report and recommendations for reform.^235^ The USA has no equivalent compulsory licensing authority within its intellectual property laws like other jurisdictions;^236^ however, the US Government does have use rights under both the *Bayh-Dole Act* and Title 28—*Judiciary and Judicial Procedure*.^237^

The *Bayh-Dole Act* was enacted to promote commercialization of government-funded research. It enabled universities and other institutions that received federal government grants to own resulting patents. However, in allocating rights in inventions developed in the course of a federal funding agreement, the US Government retained a ‘nonexclusive, nontransferrable, irrevocable, paid-up license to practice or have practiced for or on behalf of the USA any subject invention throughout the world’.^238^ In other words, the entity receiving federal funding must grant to the federal government a license in subject inventions and must notify the federal government of those inventions. In addition, the US Government has march-in rights to compel the patent holder to grant it^239^ a license under specific circumstances related to the public interest.^240^ While the NIH, in particular, has received four requests to exercise its march-in rights, it has never done so.^241^

In contrast to the *Bayh-Dole Act* that applies to federally funded inventions, *Authorization and Consent* under 28 U.S.C. § 1498 is a form of eminent domain whereby the federal government may take private property for public use subject to ‘fair compensation’ to a patent-holder.^242^ It permits the US Government, or a contractor/subcontractor acting on its behalf, to use a US patent without the consent of the patent holder. It has the effect of limiting the remedies available to the patent holder to fair compensation for the use of the invention, but excludes the most common remedies in patent infringement suits—injunctions and damages. The provision is generally implemented through the inclusion of an ‘*Authorization and Consent’* clause in Government procurement contracts, in the form specified by the *Federal Acquisition Regulations*. In this sense, the US Government provides *Authorization and Consent* to a contractor/subcontractor to use the patented invention for and on behalf of the US Government.^243^ Authorization and Consent was utilized to protect JAX in the AIA litigation and most recently to enable the US activities within an international consortium to generate a research resource of knock out mouse for every gene in the mouse genome.^244^

### Judicial action and rules for judicial proceedings

Courts, particularly in the US, have been active in debates over access to research tools. SCOTUS ruled in June 2013, in a fight over Myriad's patents on *BRCA* genes associated with hereditary risk of breast and ovarian cancer, to invalidate patent claims on DNA molecules that could be found in nature.^245^ In contrast, in September 2014, the Federal Court of Australia (FCA) unanimously upheld Myriad's *BRCA1* gene patent claim,^246^ explicitly rejecting SCOTUS’ legal logic that drew a distinction between DNA molecules ‘found in nature’ and modified cDNA molecules, which remain patent-eligible. One notable difference between the decisions was the degree to which the Courts worried about exclusive rights on information and tools needed to advance science.

While SCOTUS was primarily concerned with how patent rights might block, or ‘pre-empt’ science and upstream innovation far from commercial application, the Australian courts focused almost exclusively on the interests of prospective inventors and the value of a patent incentive to invest in discovery. The FCA mentioned detrimental effects on research only in passing, observing that Australia recently broadened its statutory research exemption from patent infringement liability as noted above.^247^ The FCA emphasized that the patent claimed not information but DNA molecules manipulated *in vitro*. SCOTUS, on the other hand, noted that the molecules in test tubes were indeed ‘isolated’, but argued that claims on such molecules in effect gave exclusive rights to block access to DNA sequences, the storage and transmission medium of biological information. The many thousands of papers published on *BRCA* mutations that use DNA molecules produced in the laboratory, and the authors of most such papers, thus infringed claims that would appear to be upheld by the Australian court.

It is apparent that the two courts relied on different mechanisms to protect research interests. The Australian court relied on the statutory protections for research outlined above, and SCOTUS, in the absence of these, focused on the definition of what can be patented to add clarity to the scope of patent claims. This is part of an ongoing trend. In addition to its unanimous *Myriad* decision, and the invalidated method claims on a diagnostic patent in *Mayo v Prometheus* (2012) noted above,^248^ SCOTUS also invalidated claims on business methods in *Bilski v. Kappos* (2010)^249^ and *Alice Corp. v. CLS Bank International* (2014).^250^ The Court of Appeals recently invalidated method and DNA molecule claims that had not been challenged in the case that went to the Supreme Court.^251^ The US Supreme Court has thus imposed constraints on patentable subject matter in a major patent case for four years in a row, and lower courts are abiding by the new jurisprudence. In response to changing SCOTUS jurisprudence, the USPTO issued guidance in March 2014 that aroused considerable controversy, and has recently been revised.^252^

The Courts may also circumvent the stall in anti-patent-troll legislation in the USA The award of fees to the winner in the present case involving AIA is an early instance of what could emerge as a powerful judicial precedent that serves as a disincentive for NPEs asserting invalid patents. New legal rules make it easier to assess costs to losers in patent suits. The federal district court thus awarded legal fees to Avid, so AIA must pay not only its costs but also fees of the defendant it sued.^253^ This measure was already on the books, but the rules for ‘exceptional circumstances’ in which they could be used were only recently loosened to address patent trolls. The former Chief Judge of the Court of Appeals of the Federal Circuit, Randall Rader, urged exactly this remedy to discourage patent suits based on invalid patents.^254^ Legal scholars have similarly touted ‘loser pays’ rules as a potent tool both to dissuade enforcement of invalid patents as well as to discourage infringement of valid patents.^255^ Proceedings to determine who pays the legal fees in *Alzheimer's Institute of America v Avid Radiopharmaceuticals* are entering the endgame supervised by a magistrate in Florida courts.^256^ One problem with this procedure is that it is largely secret. We may never know the price AIA paid for losing this case, and yet the power of the ‘loser pays’ precedent depends on those contemplating litigation knowing the risks and costs.

### Policies and guidelines

It is apparent from the *APPswe* case that institutional policies and guidelines, supported by State Law, also came into play. One reason that AIA lost its patent rights was by operation of Florida law, under which patent rights automatically vested in the USF. When patent rights are held by institutions, policies, and guidelines on how universities license inventions may significantly impact the role of NPEs.^257^ The Association of University Technology Managers recommends some caution in licensing university technologies to NPEs, for example, and recommends best practices for licensing to preserve rights for research uses.^258^ However, the guidelines are entirely voluntary, many institutions have not agreed to abide by them, and they may be trumped by increasing pressures on university technology transfer offices to generate revenue through technology licensing.

Other institutions also have significant abilities to curtail the impact of NPEs on the research community. Technically, NIH has some powerful interventions at its disposal that it rarely puts into operation: namely march-in rights under *Bayh-Dole*.^259^ Rather than employing the latter in the present case, however, the NIH employed a more obscure process available to it under the *Federal Acquisition Regulations*.^260^ *Authorization and Consent*, however, only applies to government contractors, and most researchers holding standard grants from NIH do not fall within this category. There is no case law to indicate whether this provision would apply to grants, and conventional wisdom holds that it applies only to government contractors, not grantees.

A further risk to research is aggressive licensing practices of patent holders against research uses. These were exemplified by DuPont's initial licensing terms for Oncomouse and *cre-lox* technologies in the 1990s that included reach-through terms and that imposed onerous reporting obligations on researchers. Cetus initially threatened to enforce its polymerase chain reaction (PCR) patents against academic researchers.^261^ DuPont and Roche (which acquired most rights to Cetus's PCR patents) backed down from their most aggressive licensing terms, in the former case after intervention by the NIH to negotiate access for researchers. The NIH, in other words, can apply pressure on behalf of the research community for reasonable terms of access to research tools.

### Other mechanisms

Finally, private actors are recognizing an opportunity posed by NPEs and are offering intellectual property insurance specifically directed toward the threat of suits brought by NPEs, labeled ‘Troll Defense Insurance’.^262^ Insurance, however, could be a double-edged sword. In some circumstances, it may encourage NPE suits because insurance coverage offers deep pockets to target.

## CONCLUSIONS

In conclusion, our analysis shows that in the highly competitive research environment of genetic causes for Alzheimer's disease in the 1990s, patents played a minor, subordinate role in spurring both the discovery of disease-related mutations and in creating transgenic mouse models to explore their biological mechanisms. Patent incentives did play a role in determining who hoped to make money once the discoveries were made, and here patent rights were used to control availability of some mouse models of Alzheimer's disease by Athena, Elan, and by AIA. Corporate strategies to block access to research tools were to protect in-house pharmaceutical R&D, all of which failed to product therapeutics. In other words, eggs were forced into a limited number of baskets to the detriment of scientific and clinical progress.

The scientific teams hunting for Alzheimer's genes internationally were primarily motivated by the prospect of high-impact publications in journals such as *Nature* and *Science*. Patents were secondary, and came into play once discoveries were made and published. Many of the researchers displayed considerable naiveté about patents.

The USPTO also made mistakes in prematurely granting patents on transgenic animals to someone who never developed a model, and in granting claims similar to those invalidated in *Myriad* and *Mayo*. In particular, the development of effective mouse models for Alzheimer's disease was a technologically complex undertaking, requiring intense efforts in both industry and academic research centers well beyond having the mutation sequence in hand. Academic researchers who developed mouse models were not incentivized by patents, and indeed were in some cases sued by those who did the original sequencing. Work on transgenics was motivated by scientific goals, as corroborated by free distribution networks and a focus on high-impact publications. Transgenic mouse models were made broadly available to the research community, which made distribution centers, such as Mayo and JAX, thereby vulnerable to patent infringement suits. Judge Pauline Newman raised a reasonable concern about the value of patent incentives to create transgenic animal models, but in this case, patents appear to have had the opposite effect—being used to attempt to limit distribution of animal models once they had been created, rather than as a spur to encourage their creation in the first place.

The case produces a mixed message about the patent system. It both illustrates many mistakes in how patents were obtained, administered, and enforced, but, in the end, the legal system also rectified many of these mistakes, albeit slowly, laboriously, and at great cost. It seems likely that the bulk of licensing revenue went to support the litigation much more than research. There is little public record to support AIA's contribution to research, and much legal documentation that indicates that it imposed enormous costs on the research institutions and private companies it sued. AIA's contention that it did not hamper research is not credible. AIA was thwarted from implementing its request to shut down the largest distributor of transgenic animal models of Alzheimer's disease for research because NIH entered the fray, not because of its own forbearance.

David Morgan of the USF, Tampa, a noted AD researcher who was not involved in the AIA lawsuits, observed that a flawed patent law system is the real story. ‘The lawyers are going to make money no matter what happens. Whether AIA wins or loses, there are lawyers everywhere who benefit. There's no productive value in what they're doing.’ Another scientist who refused identification for fear of being drawn into pending AIA suits, ‘This has got to be one of the nastiest things that has happened in the AD field.’ Nevertheless, this same scientist noted Mullan ‘legitimately got these patents. I don't fault the guy for insuring his intellectual property is safeguarded. The issue is how much is legitimate policing of IP and how much is disregard for scientific progress’.^263^ Given the public record, this concedes too much.

AIA clearly puts research institutions in its litigation crosshairs and hampered Alzheimer's research in order to extract money without significantly contributing to the field or advancing delivery of a service or therapeutic. The legal system ultimately neutered AIA's ability to continue these practices. This is nonetheless a cautionary tale that broad exclusive patent rights applied to research tools can (and did) become real-world impediments to biomedical research.

## EPILOG: WHERE ARE THE KEY ACTORS NOW?

Elan Pharmaceuticals: in January 2002, Elan became embroiled in an accounting scandal. Its stock prices sank to ${\$ }$14.80, and an investigation was launched by the US Securities and Exchange Commission. This company that was once the most capitalized company in Ireland, had a historic fall from its peak of 50.27–16.50 Euros on the Irish stock exchange. By April, Elan's shares were worth ${\$ }$11.32 on the New York Stock Exchange. Later in the decade, Elan recovered somewhat, but in 2010, Elan was fined ${\$ }$203 million for its marketing of epilepsy drugs. On December 18, 2013, Elan was acquired by Perrigo Company PLC for approximately ${\$ }$8.6 billion USD.^264^ Perrigo is a global healthcare supplier of over-the-counter and generic prescription pharmaceuticals, nutritional products, and active pharmaceutical ingredients.

Alzheimer's Institute of America: the AIA retains a website with tagline ‘Supporting Brain Research Worldwide’ with a copyright date of 2009.^265^ All links on research, technology, commercial and academic licensing opportunities, and funding opportunities request viewers to ‘please check back later’ because AIA is working ‘to update our website and anticipate having the information you've requested available online soon’. The site posts two initiatives—a global family initiative for patients and families and a research initiative with grants, fellowships, and a research portal, none of which are active.

AIA Foundation: the Foundation website has the tagline ‘Sharing Memories for a Lifetime’.^266^ Its last blog entry is dated August 16, 2012. The website states that the Foundation was founded in 1992 but that is founding date for AIA, the Foundation was founded in 2010. The donation pages remain active. There is no evidence available of projects funded by the AIA Foundation.

Brian Sexton: Mr Sexton is currently involved in litigation over real estate.

John Hardy: Hardy returned to the UK in 2007 and is now a Professor of Neuroscience at University College London after a period at the National Institute of Aging, Bethesda, Maryland. He has over 23,000 citations and is the most cited Alzheimer's disease researcher in the UK (5 internationally).^267^ He was elected a Fellow of the Royal Society in 2009.

Michael Mullan: Mullan's highest impact publications were the result of his collaboration with Hardy. In the 1997–2002 time period, after Hardy left USF, Mullan published 78 articles, with one in *Science* and one in *Nature Neuroscience*, both highly cited and both with Mullan as senior author.^268^ In 1996, he had a senior authored paper in *Nature* which has been cited 502 times. While Mullan is still referred to in newspaper reports as a leading Alzheimer's disease researcher, since leaving USF for the Roskamp Institute in 2003, he has published 90 articles, none in top-tier journals such as *Science* and *Nature*. This may reflect a focus on commercialization. He is now the CEO of Star Scientific Inc. under the new name of Rock Creek Pharmaceuticals.

Star Scientific Inc. and Rock Creek Pharmaceuticals: Star Scientific Inc., was a dietary supplements company ‘whose former top executive was embroiled in a gift-giving scandal with former [Virginia] Gov. Bob McDonnell’.^269^ The company moved to Sarasota, Florida. The company was a former maker of discount cigarettes that switched to alternative tobacco products followed by dietary supplements. The company now states that its focus is on pharmaceutical development for Alzheimer's disease. The *Herald Tribune* reported that the Roskamp Institute was interested in taking over an anti-inflammation drug that had shown promise and that had been developed by Star Scientific. The new company plans clinical trials and Mullan believes that following FDA approval, the company could be an acquisition target for a large pharmaceutical company, such as Pfizer, Eli Lilly & Co., or GlaxoSmithKline (note ironically that AIA had sued the first 2 over patents held by Mullan).^270^ Star Scientific had, however, run afoul of the FDA by making inappropriate health claims for one of its dietary supplements (over-the-counter drugs). To counter the unfavorable publicity, Mullan as CEO changed the name to Rock Creek Pharmaceuticals, Inc., Star had developed a prescription version derived from anatabine, an alkaloid present in tobacco, tomatoes, and eggplants. The compound is sold for anti-inflammatory effects and Alzheimer's disease. Mullan owns stock options on six million shares. One commentator stated his guess ‘that there will be conversations with a major pharmaceutical partner going on over the next two years, and that a deal of some sort will be struck’.^271^

Roskamp Institute: the Roskamp Institute is listed as collaborator with Rock Creek, Pharmaceuticals, Inc., and as sponsor of one completed clinical trial for a dietary supplement, Anatabine (NCT01607619) and a second suspended study of a second dietary supplement, Anatabloc in Alzheimer's disease (NCT01669876).^272^ The completed study was last verified in January 2013, after approval in February 2012; the clinical trial sponsor was the Roskamp Institute. However, no results were posted as of December 2014 from this trial which aimed to enroll 117 healthy adults (not adults with Alzheimer's disease). The listed investigators did not include Mullan. A second study was also listed under another investigator, to enroll 200 people aged 65–90.

## NOTE ADDED IN PROOF

On March 30, 2015, Judge Savage issued an order on fee shifting.^273^ In addition, he appointed a Special Master to report and recommend on the reasonable attorney fees incurred by the defendants in the litigation.^274^ In issuing his order on fees, Judge Savage applied *Octane*'s interpretation of Section 285 of the *Patent Act*, which allows attorney's fees to be awarded in an “exceptional case”.^275^ *Octane* held that “an exceptional case is simply one that stands out from others with respect to the substantive strength of a party's litigating position … or the unreasonable manner in which the case was litigated”.^276^ Judge Savage described how AIA is such an exceptional case. He stated “The evidence at trial clearly supports our findings that Sexton, Hardy and Mullan conspired to misrepresent the true inventorship of the Swedish mutation inventions in an effort to ensure that ownership of those inventions could not be claimed by Imperial College; and that they intentionally hid the discovery of the mutation from USF to avoid its claiming rights in the invention.”^277^ He concluded by stating “The deception, the planning, the execution of the scheme and the motivation of AIA, Sexton, Mullan and Hardy were hardly common or ordinary. Indeed, their conduct was rare and beyond common decency. They were motivated by ego and greed. Bringing this action was nothing more than a perpetuation of the conspiracy.”^278^ He determined that “AIA should be held responsible for its bad faith and unreasonably bringing an action for infringing a patent that was unenforceable.”^279^

## Footnotes

1 686 F.3d 1348, 1375 (Fed. Cir. 2012) at 1375 (summarizing academic literature and policy reports that find a lack of data to support the anticommons hypothesis at).

2 *Id.* (citing Wesley M. Cohen & John P. Walsh, *Real Impediments to Academic Biomedical Research, in* 8 Innovation Pol'y & Economy 1, 12 (Adam B. Jaffe et al. eds., 2008), http://www.nber.org/chapters/c5300.pdf) (accessed Mar. 25, 2015).

3 *Id.* at 1376.

4 We examined the acknowledgements section in 197 publications authored by Michael Mullan accessible in the SCOPUS database (http://www.scopus.com) from 1992 to 2014. We found no record of acknowledgement of the Alzheimer's Institute of America or its principals. On the other hand, Mr and Mrs Roskamp and their research foundation were acknowledged in a substantial number of publications dating from 1995. Other funding came from pharmaceutical and biotechnology companies, state and federal funding agencies, disease foundations, and the US Government.

5 See Mayo Collaborative Services v. Prometheus Lab., 566 U.S. 10 (2012); Association for Molecular Pathology v. Myriad Genetics, *133 S. Ct. 2107* (2013); Bilski v. Kappos, 561 U.S. 593 (2010); Alice Corp. Pty. Ltd. v. CLS Bank Intern., *134 S. Ct. 2347* (2014). But see also *infra* note 223 on fee-shifting and injunctions.

6 The key facts are laid out in two decisions by Judge Savage of the US District Court, East District, Pennsylvania: Alzheimer's Inst. of America, Inc. v. Avid Radiopharmaceuticals, No. 10-6908, 2011 WL 3875341 (E.D., Pa. Aug. 31, 2011), http://www.paed.uscourts.gov/documents/opinions/11d0989p.pdf (accessed Dec. 22, 2014); Alzheimer's Inst. of America, Inc. v. Avid Radiopharmaceuticals, 952 F. Supp. 2d 740, 2013 WL 3305738 (E.D. Pa. 2013), http://docs.justia.com/cases/federal/district-courts/pennsylvania/paedce/2:2010cv06908/398886/345 (accessed Dec. 23, 2014).

7 Alzheimer's Association, *Major Milestones in Alzheimer's and Brain Research* (2014), http://www.alz.org/research/science/major_milestones_in_alzheimers.asp (accessed Dec. 11, 2014): the greatest risk factor for developing Alzheimer's disease is age, with a lifetime risk of developing Alzheimer's estimated at 26.3, 28.7, and 32.4 per cent for individuals over the age of 65, 75, and 85, respectively. Its symptoms are progressive, starting with difficulty remembering conversations, names and events and moving to impairments in communication, disorientation, confusion, poor judgment, and behavior changes. Eventually, these lead to difficulty in speaking, swallowing, and walking.

8 Id.

9 Medicines in Development, *Biopharmaceutical Research Companies Are Developing Nearly 75 Medicines for Alzheimer's Disease*, at 1–12 (2013), http://www.phrma.org/sites/default/files/Alzheimer%27s%202013.pdf (accessed Dec. 22, 2014) [hereinafter, *Medicines in Development*] .

10 Nilufer E. -Taner, *Alzheimer Disease: The Quest for Alzheimer Disease Genes - Focus on SCF Tau*, 9 Nat. Rev. Neurol. 368, 368–79 (2013).

11 Allen D. Roses, *On the Discovery of the Genetic Association of Apolipoprotein E Genotypes and Common Late-Onset Alzheimer Disease*, 9 J Alzheimer's Dis. 361, 366 (2006). Those who inherit two copies of the ε4 allele have an odds ratio of 16 relative to the population; but presence of one or two copies of the allele is neither necessary nor fully predictive of the development of Alzheimer's disease.

12 The average age of onset is 52 years, with a range of onset from 40–75 years. The age of onset of Alzheimer's for both *PSEN* genes is earlier if also associated with the APOE ε4 allele.

13 Indeed, only about 30 known families worldwide have mutations in the *APP* gene, located on chromosome 21. The *APP* gene contains at least 30 mutations that cause EOAD in individuals from 39 to 67 years of age. There are about 173 pathogenic mutations of *PSEN1*, located on chromosome 14. Individuals with these mutations have severe clinical symptoms with onset between 28 and 64 years of age (average age of onset is 45 years). The *PSEN1* gene, which has 14 pathogenic mutations, was found in American descendants of German families that had previously settled in the Volga River region of Russia. These mutations have only been discovered in this one familial group.

14 Alzheimer's Association, *supra* note 7.

15 Eg Eric Karran & John Hardy, *Antiamyloid Therapy for Alzheimer's Disease - Are We on the Right Road?*, 370 New Eng. J. Med. 377, 378 (2014); Eric Karran et al., *The Amyloid Cascade Hypothesis for Alzheimer's Disease: An Appraisal for the Development of Therapeutics*, 10 Nat. Rev. Drug Disc. 698, 698–712 (2011).

16 Intermediary forms of the peptide possibly account for the neurotoxicity of the amyloid-β peptide, suggesting that mutations in other enzymes that clear amyloid- β or cleave APP might also be involved. For example, the APOE ε4 isoform of apolipoprotein that breaks down amyloid-β is less efficient than other isoforms. In addition, a specific fragment called N-APP, which is adjacent to amyloid- β and is cleaved from APP by the same enzymes, triggers neuronal death and complements amyloid- β that depresses synaptic function.

17 From 1998 to 2011, there were 101 drugs in clinical trials, but only three new medicines were approved to treat symptoms of Alzheimer's disease, not its underlying pathophysiology (*Medicines in Development, supra* note 9, at 1). In total, the US Food and Drug Administration has approved five medications to treat the symptoms of Alzheimer's disease: tacrine (Cognex; 1993); donepezil (Aricept; 1996), rivatigmine (Excelon; 2000), galantamine (Razadyne; 2001); and memantine (Namenda; 2003). Donepezil is indicated for all stages of the disease, memantine for moderate to severe stages, and the rest for mild to moderate Alzheimer's disease. Alzheimer's Association, *What We Know today About Alzheimer's Disease*, 2014, http://www.alz.org/research/science/alzheimers_disease_treatments.asp (accessed Dec. 16, 2014).

18 Such novel approaches include targeting of upstream pathways, gene therapy to restore neuronal function, boosting immune responses with vaccine treatments, prevention of plaque formation, and inhibition of amyloid-β production (*Medicines in Development, supra* note 9, at 2).

19 Rudolph E. Tanzi & Ann B. Parson, Decoding Darkness: The Search For The Genetic Causes Of Alzheimer's Disease 177 (2000). (Citing John Hardy). Another gene hunt illustrative of the degree of competition in the field at the time was the hunt for the EOAD gene on chromosome 14. The linkage to chromosome 14 was simultaneously published by the Schellenberg team in *Science* (Gerard D. Shellenberg et al., *Genetic Linkage Evidence for a Familial Alzheimer's Disease Locus on Chromosome 14*, 258 Science 668, 671 (1992)) and by three teams in three papers in *Nature Genetics* (Mike Mullan et al., *A Pathogenic Mutation for Probable Alzheimer's Disease in the APP Gene at the N-terminus of β–amyloid*, 2 Nat. Genet. 345, 347 (1992); Peter St George-Hyslop et al., *Genetic Evidence for a Novel Familial Alzheimer's Disease Locus on Chromosome 14*, 2 Nat. Genet. 330, 334 (1992); Christine Van Broeckhoven et al., *Mapping of a gene predisposing to early-onset Alzheimer's disease to chromosome 14q24.3*, 2 Nat. Genet. 335, 339 (1992). According to Tanzi & Parson, *supra* note 19, at 151, there were rumors of piggybacking given the timing of the papers. Van Broeckhoven claimed that she first identified the linkage but was waiting to identify the gene. Hearing of the other papers, she raced to print. However, ‘the most rumor-driven collision was between Hyslop and the Hardy-Mullan team’ (Tanzi & Parson, *supra* note 19, at 152). St George-Hsylop's paper was submitted to *Nature* and sent to the Hardy/Mullan team, among others, for review. They faulted it on the basis of St George-Hyslop's prior linkage in the same families to chromosome 21; it was highly unlikely that the individuals carried two mutations. *Nature* rejected the paper ‘and Hyslop, rumor had it, felt that Hardy and Mullan had delayed his paper's publication for the sake of completing their own chromosome 14 paper’ (Tanzi & Parson, *supra* note 19, at 152). Three years later, however, St George Hyslop led the team that cloned the presenilin gene on chromosome 14, winning a tight race with the Tanzi, Schellenberg, and the Hardy/Van Broeckhoven teams(Robin Sherrington et al., *Cloning of a gene bearing missense mutations in early-onset familial Alzheimer's disease*, 375 Nat. Genet. 754, 760 (1995)).

20 *Id.* and Daniel A. Pollen, Hannah's Heirs: The Quest for the Genetic Origins of Alzheimer's Disease (exp. sub. ed. 1996) (1993).

21 *Id*.

22 *Id*. For example, Peter St George-Hyslop, then a Ph.D. student with James Gusella at the Massachusetts General Hospital, had access to a growing collection of samples of Alzheimer's families. Initially he focused on a British Canadian family, described by Linda Nee and Ronald Polinsky at the National Institutes of Health in 1983 and a large family of Southern Italian ancestry provided by Rick Myers and collected by Robert Feldman at Boston University. Daniel A. Pollen at the University of Massachusetts University Medical Centre studied a Russian-American family and, in Seattle, Gerard Schellenberg studied a Volga German family, whose samples and pedigree had been collected by Thomas Bird at the University of Washington. In Europe, Christine Van Broeckhoven studied Belgian families; Jean-Francois Foncin and V. Supino-Viterbo found a French family; and John Hardy's group had a newly identified British family (*Id.* at 206). In 1985, combined data from the French and Italian families and their American descendants showed a common ancestor: Jean Francois Foncin et al., *Demence Presenile d'Alzheimer Tranmise Dans Unie Famille Etendue*, 141 Revue Neurologic 194, 194–202 (1985).

23 George G. Glenner & Caine W. Wong, *Alzheimer's Disease: Initial Report of the Purification and Characterization of a Novel Cerebrovascular Amyloid Protein*, 122 Biochem. Biophys. Res. Commun. 1131, 1131–35 (1984). Accumulation occurs in the walls of small cerebral blood vessels. Individuals with Down syndrome also display ‘senile plaques’ in their brains.

24 Rudolf Tanzi was then a Ph.D. student in the lab of Dr James Gusella at the Massachusetts General Hospital.

25 Tanzi & Parson, *supra* note 19, at 49–59. Chromosome 21 is the shortest autosomal human chromosome.

26 Peter St George Hyslop, *supra* note 22.

27 Especially in a large Italian family.

28 The linkage to this region close to the centromere on Chromosome 21 that contained the *APP* gene was calculated with a significant LOD score for genetic linkage. St George-Hyslop et al. *The Genetic Defect Causing Familial Alzheimer's Disease Maps on Chromosome 21.* 235 Science 885, 885–90 (1987). LOD stands for logarithm of the odds. It is ‘a statistical estimate of whether two genes, or a gene and a disease gene, are likely to be located near each other on a chromosome and are therefore likely to be inherited. A LOD score of 3 or higher is generally understood to mean that two genes are located close to each other on the chromosome. In terms of significance, a LOD score of 3 means that the odds are a thousand to one that the two genes are linked, and therefore inherited together': Lawrence C. Brody, *National Human Genome Research Institute. Talking Glossary of Genetic Terms*, http://www.genome.gov/Glossary/index.cfm?id=531 (accessed Dec. 18, 2014).

29 Tanzi & Parson, *supra* note 19, at 49–59 and Dmitry Goldgaber et al., *Characterization and Chromosomal Localization of a cDNA Encoding Brain Amyloid of Alzheimer's Disease*, 235 Science 877, 877–80 (1987) .

30 Jie Kang et al., *The Precursor of Alzheimer's Disease Amyloid A4 Protein Resembles a Cell-Surface Receptor*, 325 Nature 733, 733–36 (1987).

31 Peter St George-Hyslop*, supra* note 22. St George-Hyslop was at Massachusetts General Hospital; Christine Van Broeckhoven et al., *Failure of Familial Alzheimer's Disease to Segregate with the A4-Amyloid Gene in Several European Families*, 329 Nature 153,155 (1987). Van Broeckhoven and colleagues were at University of Antwerp, Belgium.

32 Pollen, *supra* note 20, at 153.

33 Hereditary cerebral hemorrhage with amyloidosis of the Dutch type. The Dutch disease causes a similar plaque buildup to Alzheimer's, but in the brain vasculature: Orphanet, *Hereditary cerebral hemorrhage with amyloidosis, Dutch type*, http://www.orpha.net/consor/cgi-bin/OC_Exp.php?Expert=100006 (accessed Dec. 22, 2014).

34 The two teams were led by Christine Van Broeckhoven and Blas Frangione of New York University. Frangione sequenced the nucleotide substitution, locating it on exon 17. Efrat Levy et al., *Mutation of the Alzheimer's Disease Amyloid Gene in Hereditary Cerebral Hemorrhage, Dutch Type.* 248 Science 1124, 1124–26 (1990); Christine V. Broeckhoven et al. *Amyloid Beta Protein Precursor Gene and Hereditary Cerebral Hemorrhage with Amyloidosis (Dutch)*, 248 Science 1120, 1120–22 (1990).

35 Approximately 70 base pairs from the Dutch disease mutation.

36 Marie-Christine Chartier-Harlin et al., *Early-Onset Alzheimer's Disease Caused by Mutations at Codon 717 of the Beta-Amyloid Precursor Protein Gene*, 353 Nature 844, 844–46 (1991).

37 Allen Roses from Duke University was best-known for identifying the APOE gene.

38 Chartier-Harlin, *supra* note 36.

39 Pollen, *supra* note 20, at 213.

40 Tanzi & Parson, *supra* note 19, at 120.

41 *Id.* at 135. See also Pollen, *supra* note 20, at 213.

42 Most large groups did not find the mutation, including the group at Massachusetts General Hospital and Schellenberg's group in Seattle.

43 In this context, ‘false’ means that the conclusions drawn from the data were incorrect, not that the data were deliberately falsified.

44 Tanzi & Parson, *supra* note 19, at 129. But Tanzi's use of the term ‘false’ is not intended to imply fabrication, but rather that the data turned out not to determine linkage between mutations on Chromosome 21 and Alzheimer's disease.

45 Alzheimer's Inst. of America, 2011 WL 3875341, *supra* note 6, at 2.

46 Id.

47 US Patent No. 5877,015 (filed Jan. 21, 1992).

48 Alzheimer's Inst. of America, 2011 WL 3875341, *supra* note 6, at 4.

49 Id.

50 UK Patents Act, 1977, c. 37, § 39.

51 Catherine Hollingsworth, *Archer Pharma Working on New Version of AD Drug*, BioWorld Today, 2009, at 8., http://www.bioworld.com/content/archer-pharma-working-new-version-ad-drug (accessed Dec. 22, 2014).

52 Alzheimer's Inst. of America, 2011 WL 3875341, *supra* note 6, at 3.

53 *Id.*; Alzheimer's Inst. Of America, 2013 WL 3305738, *supra* note 6, at 3.

54 Esther Landhuis, *Court Ruling Sinks APPswe Patent Suit Claims*, AlzForum 2012, http://www.alzforum.org/news/community-news/court-ruling-sinks-appswe-patent-suit-claims (accessed Dec. 22, 2014).

55 Alzheimer's Inst. of America, 2011 WL 3875341, *supra* note 6, at 5, 6.

56 Mullan et al., *supra* note 19.

57 Alzheimer's Inst. of America, 2011 WL 3875341, *supra* note 6, at 3.

58 Publications co-authored by Mullan from 1989 to 1995 and listed in PubMed identified the following funding sources: The Medical Research Council; The Mental Health Foundation; Research into Ageing; The Foundation for Age Research; The Royal Society; Merck Sharpe and Dohme Ltd; Wellcome Trust; NATO; Alzheimer's Disease Society; The IPSEN Foundation; The Royal Society; and Athena Neuroscience. Other funding sources were the Howard Hughes Medical Institute which supported the work of co-author L. Jan at the University of California San Francisco. Swedish source of funding came from the Swedish Medical Research Council and Tore Nilson's Fund. Work in 1995 acknowledged the Texas Department of Health DNA bank for genetics studies of Alzheimer's disease and Robert Roskamp.

59 Alzheimer's Inst. of America , 2013 WL 3305738, *supra* note 6.

60 *Id.* at 4.

61 Mullan and Crawford received their Ph.D. degrees in 1993 and 1994, respectively.

62 Imperial College current IP policy states: the Patents Act 1977 and the Copyright, Designs and Patents Act 1988, with any related IP legislation, make it clear that all forms of IP generated by an employee, made in the course of the employee's normal duties, belong to their employer. Hence, as prescribed by law, any IP created by College employees in the course of their normal duties shall be the property of College. (See Imperial College, *Intellectual Property Policy*, Jul. 15, 2009, https://workspace.imperial.ac.uk/researchservices/Public/College%20IP%20Policy%20public%20access.pdf (accessed Dec. 19, 2014).

63 Alzheimer's Inst. of America , 2011 WL 3875341, *supra* note 6.

64 *Id.* at 6.

65 *Id.* at 4.

66 US Patent No. 5455,169 (filed Jun. 4, 1992). It claimed the nucleic acid of the human amyloid precursor protein comprising the Swedish mutation, a vector that includes the Swedish mutation and an immortalized mammalian cell line that contains the Swedish mutation.

67 US Patent No. 7538,258 (filed Mar. 8, 2007) [hereinafter ‘258 patent’]. A continuation patent uses the same disclosure specification as the initial or parent application, claims the priority date of the parent patent, and generally names at least one of the same inventors. It is often used when an applicant feels he/she has not claimed all of the different embodiments of the invention in the parent patent. See discussion on strategic issuance delay, *infra* note 205, at 298–301.

68 Tanzi & Parson, *supra* note 19, at 162.

69 *Id* at 163.

70 Id.

71 Id.

72 Rex Dalton et al., *Researchers Caught in Dispute Over Transgenic Mice Patents*, 40 Nature 319 (2000).

73 Id.

74 Id.

75 Roskamp Institute, *Scientific Research at the Roskamp Institute*, http://www.rfdn.org/ (accessed Dec. 12, 2014).

76 Hollingsworth, *supra* note 51, at 8.

77 The company is listed as a collaborator in a phase III human clinical trial for the re-purposing of the blood pressure drug Nilvadipine, sponsored by researchers at Trinity College Institute of Neuroscience, Dublin, Ireland (recruiting 2013, NCT02017340; clinicaltrials.gov). The Trinity College trial is the only clinical trial that lists Archer Pharmaceuticals as Collaborator (or Sponsor) registered on Clinicaltrials.gov. The trial was funded in 2011 by a Six million Euro grant from the European Commission's Framework Seven Programme. Sharon Campbell, *Clinical Trials for New Alzheimer's Disease Treatment Led by Trinity College Dublin Researchers to be Awarded European Commission €6 million Funding*, 29 May 2011, https://www.tcd.ie/Communications/news/pressreleases/pressRelease.php?headerID=1856&pressReleaseArchive=2011, (accessed Dec. 23, 2014).

78 Guidestar, 2014, ‘Alzheimer's Institute of America’, http://www2.guidestar.org/ (accessed Dec. 11, 2014).

79 Alzheimer's Institute of America Inc., *Supporting Brain Research Worldwide*, 2009, http://www.alzheimersinstitute.com/ (accessed Dec. 11, 2014).

80 On Oct. 9, 2014, we searched the NCBI Gene database (National Center for Biotechnology Information, http://www.ncbi.nlm.nih.gov/gene) for the *APP* gene. We then selected all 1939 citations in the PubMed database (US National Library of Medicine, National Institutes of Health, http://www.ncbi.nlm.nih.gov/pubmed) linked to the *APP* gene record in the NCBI Gene database. We exported these in XML format and parsed out the PubMed IDs (unique publication identifiers). We then searched a second proprietary publication database, SCOPUS (Elsevier, Scopus, http://www.elsevier.com/online-tools/scopus) for the PubMed IDs, because the data it returns are in a format more amenable to bibliometric analyses. We constructed a dataset of *APP* gene publications (*n* = 1869). We disambiguated the authors’ names by hand, ensuring that authors did not appear more than once, if, for example, they had publications using either their first or first and middle initial. We used Gelphi (http://gephi.github.io/) to visualize the co-authorship linkages and generate the figures. Gelphi uses graph theory to visualize relationships between authors (Mason A. Porter et al., *Communities in Networks*, 56 Notices Am. Mathematical Soc'y 1082 (2009)). It assigns a unique node to each author. The lines between nodes reflect the relationship between them. Gelphi identifies communities of authors (represented by different colors) using algorithms that identify discrete groups of nodes with dense connections internally, but sparse connections between groups (Kyoungok Kim, *Improved Churn Prediction In Telecommunication Industry by Analyzing a Large Network.* 41 Expert Sys. Applications 6575 (2014)).

81 *Id.* We used the same method to generate Figure 2 as Figure 1 with a date range of 2000 to October 2014.

82 Lex Machina, Data compiled from Lex Machina, https://law.lexmachina.com/cases/15497 (accessed Dec. 23, 2014).

83 *Id.* Lex Machina Results. Alzheimer's Inst. of America v. Mayo Clinic, No. 03–02645. (2005).

84 AIA filed a motion to seal the complaint due to confidential information, which motion was granted on Dec. 19, 2003 (Pl. Mot. to Seal Compl., No. 03–02645, Dec. 19, 2003). |AIA requested the trial to take place in Kansas City, the location of its office, and the home of Sexton. After 40 separate docket entries of motions, filings, orders, pleadings etc. and over a year and a half, the case was transferred from the District of Kansas to the Middle District of Florida on 6 June 2005 (Alzheimer's Inst. of America, No. 03–02645, (2005)). There it accumulated a further 28 docket entries over 7.6 years until it ended.

85 Esther Landhuis & Gabrielle Strobel, *Law and Disorder - APPswe Patent Suits Raise Ruckus Again*, AlzForum 2010, http://www.alzforum.org/news/community-news/law-and-disorder-appswe-patent-suits-raise-ruckus-again (accessed Dec. 23, 2014).

86 Alzheimer's Inst. of America , 2011 WL 3875341 and , 2013 WL 3305738, *supra* note 6.

87 Dalton, *supra* note 73.

88 Brian Sexton, (posted Jun 22, 2010), comment in response to Landhuis & Strobel, *supra* note 83.

89 346 F.3d 1051 (Fed. Cir. 2003).

90 Funding Universe, *Elan Corporation PLC History, International Directory of Company Histories*, 2004 http://www.fundinguniverse.com/company-histories/Elan-Corporation-PLC-Company-History.html (accessed Dec. 23, 2014).

91 BioPharmaLink, *Athena Diagnostics (Athena Neurosciences Inc.)* (2002) http://www.biopharmalink.com/companies/1677.htm (accessed Dec. 15, 2014).

92 Dalton, *supra* note 73.

93 Id.

94 Karen Hsiao et al., *Correlative Memory Deficits, Abeta Elevation, and Amyloid Plaques in Transgenic Mice*, 274 Science 99 (1996).

95 Taconic, *Taconic Biosciences*, 2014, http://www.taconic.com/?gclid=COygi5ySm8ECFZSPfgodwxAAOg (accessed Dec. 18, 2014).

96 Steven G. Younkin & Susan Stoddard, *Mayo and the Mouse*, 404 Nature 809, 809 (2000).

97 *Id*.

98 Dalton, *supra* note 72.

99 Elan Pharm., Inc. v. Mayo Foundation for Med. Educ. and Research, 175 F. Supp. 2d 1209 (N.D.Cal.2000)

100 35 U.S.C. § 102(a) (2012). To be relevant for novelty purposes, the disqualifying prior art must disclose the invention in a manner that enables a person of ordinary skill in the art, without undue experimentation, to make or put it into practice.

101 Rex Dalton, *Patent Suit on Alzheimer's Mouse Rejected*, 405 Nature 989, 989 (2000).

102 Elan Pharm., Inc. v. Mayo Foundation, Med. Educ. and Research*.*, 304 F.3d 1221 (Fed. Cir. 2002).

103 *Id.* at 1228.

104 Id.

105 *Id.* at 1229.

106 Elan Pharmaceuticals, Inc. and Athena Neurosciences, Inc. v. Mayo Foundation for Medical Education and Research, 346 F.3d. 1051 (2003). Rule 35 of the *Federal Rules of Appellate Procedure* determines when an *en banc* (one before a majority of the circuit judges who are in regular active service and are not disqualified) hearing or rehearing is warranted. It is only ordered is ‘(1) en banc consideration is necessary to secure or maintain uniformity of the court's decisions; or (2) the proceeding involved a question of exceptional importance’. Cornell University Law School. *Federal Rules of Appellate Procedure*, http://www.law.cornell.edu/rules/frap/rule_35 (accessed Dec. 16, 2014).

107 *Id.*, citing Minnesota Min. and Mfg. Co. v. Chemque, Inc, 303 F.3d 1294 (Fed. Cir. 2002); Enzo Biochem. Inc. v Calgene Inc., 188 F.3d 1362 (Fed. Cir. 1999).

108 Specifically, of: ‘(1) the quantity of experimentation necessary, (2) the amount of direction or guidance presented, (3) the presence or absence of working examples, (4) the nature of the invention, (5) the state of the prior art, (6) the relative skill of those in the art, (7) the predictability or unpredictability of the art, and (8) the breadth of the claims’. *Id.* at ¶ 24; citing factors from in re Wands, 858 F.2d 731, 8 USPQ 2d 1400 (Fed. Cir. 1988).

109 *Id*.

110 *Id*.

111 Tom Fagan, *Hushing the Mouse that Roared—Elan Mayo Patent Dispute Settled*, AlzForum 2004, http://www.alzforum.org/news/community-news/hushing-mouse-roared-elan-mayo-patent-dispute-settled (accessed Dec. 23, 2014).

112 To be sure, an earlier patent such as Mullan's ‘258 patent may be sufficiently (and patentably) enabling with respect to what it claims—while not enabling so much as to inherently anticipate (and invalidate) a later patent such as Elan's. In this case, however, Mullan was also asserting rights over the transgenic mice that Elan was claiming. For that reason, enablement of Mullan's ‘258 patent as anticipatory prior art toward Elan's patent does, indeed, also reflect enablement of Mullan's ‘258 patent as a condition of patentability. If Mullan's ‘258 patent was enabled, Elan's patent rights cannot survive, and if Elan's patent rights over transgenic mice are legitimate, then Mullan's ‘258 patent was likely not patentably enabled to the extent of covering transgenic mice.

113 Fagan, *supra* note 109.

114 International Mouse Strain Resource (IMSR), http://www.findmice.org (accessed Nov. 19, 2014).

115 Bruce Boman, *Of Mice and Men: a Curious Tale of Disappearing Plaques and Tumbling* S*tocks*, 10 A. Psychiatry, 287, 287–89 (2002), at 288, http://onlinelibrary.wiley.com/doi/10.1046/j.1440-1665.2002.00465.x/abstract (accessed Dec. 23, 2014).

116 Dalton, *supra* note 72.

117 Dale Schenk et al., *Immunization with Amyloid-[beta] Attenuates Alzheimer-Disease-like Pathology in the PDAPP Mouse*, 400 Nature 173, 173–77 (1999).

118 Boman, *supra* note 113, at 288.

119 Id.

120 Id.

121 A recent article in *Nature Biotechnology* indicates that only 15.3 per cent of investigational drugs gain regulatory approval in their lead indication and only 10.4 per cent in all indications. The attrition from phase 1–2 in lead indications is 33 per cent, from phase 2–3 is 61 per cent. Michael Hay et al. *Clinical Development Success Rates for Investigational Drugs.* 32 Nat. Biotechnol 40 (2014).

122 As previously discussed, if Mullan's ‘258 patent were enabled and valid, then Elan's patents on transgenic mice could not be, meaning that at least Elan's patents provided no incentive for invention of transgenic mice as a research tool, International Mouse Strain Resource (IMSR), *supra* note 112.

123 Landhuis & Strobel, *supra* note 83.

124 Lex Machina Results. Alzheimer's Inst. of America, Inc. v. Elan Corp. P.L.C., Order Den. Leave to Amend, Mar. 16, 2011, No. 10–00482. https://law.lexmachina.com/cases/200000190.

125 Id.

126 Id.

127 Francis S. Collins. *NIH Director's Statement on Lawsuit Related to Mouse Models of Alzheimer's Disease*, NIH, Aug. 11, 2011, http://www.nih.gov/about/director/08122011_statement_alzheimers.htm (accessed Dec. 23, 2014).

128 Tania Bubela et al., *Managing Intellectual Property to Promote Pre-Competitive Research: The Mouse as a Model for Constructing a Robust Research Commons*, 20 J. L. Info. & Sci. 98, 98–121 (2012); Paul N. Schofield et al., *Post-Publication Sharing of Data and Tools*, 461 Nature 171, 171–73 (2009).

129 Order Den. Leave to Amend, No. 10–00482, Mar. 16, 2011.

130 Id.

131 Id.

132 Landhuis & Strobel, *supra* note 83.

133 Id.

134 *Id.* [Emphasis added]

135 Landhuis & Strobel, *supra* note 83.

136 *Id.*; Erika Check Hayden, *Patent Dispute Threatens US Alzheimer's Research*, 472 Nature 20 (2011). Sexton response in Nature comments.

137 Id.

138 Id.

139 The most recent milestone in patent law's experimental use doctrine was effectively its abolition in Madey v. Duke Univ., 307 F.3d 1351 (2002), but the literature long before recognized the problem of distinguishing academic versus commercial (or basic versus applied) research for purposes of patent infringement analysis. See Rebecca S. Eisenberg, *Patents and the Progress of Science: Exclusive Rights and Experimental Use*, 56 U. Chi. L. Rev. 1017, 1018 (1989) (arguing that economic trends in biotechnology research had blurred the distinction between basic and applied research); Rebecca S. Eisenberg, *Proprietary Rights and the Norms of Science in Biotechnology Research*, 97 Yale L.J. 177, 195–97 (1987) (same). Indeed, despite the demise of experimental use in *Madey*, debate over the distinction remains a live issue. See Katherine J. Strandburg, *User Innovator Community Norms: At the Boundary Between Academic and Industry Research*, 77 Fordham L. Rev. 2237 (2009).

140 For two particularly influential discussions in the legal literature of the role that patents should play in translating upstream academic research into downstream market goods, *compare* Arti K. Rai, *Regulating Scientific Research: Intellectual Property Rights and the Norms of Science*, 94 Nw. U. L. Rev. 77 (1999), *with* F. Scott Kieff, *Property Rights and Property Rules for Commercializing Inventions*, 85 Minn. L. Rev. 697 (2001).

141 Casey McDonald, *Alzheimer's Mouse Model Lawsuit Dismissed*, BioTechniques (2011), http://www.biotechniques.com/news/Alzheimers-Mouse-Model-Lawsuit-Dismissed/biotechniques-320967.html (accessed Dec. 23, 2014).

142 Check Hayden, *supra* note 134; Landhuis & Strobel, *supra* note 83.

143 Landhuis & Strobel, *supra* note 83.

144 Copy of letter on file with the authors.

145 Id.

146 Pursuant to 28 U.S.C. § 1498 (2006) and ‘*Authorization and Consent*’ under the *Federal Acquisition Regulations* Clause 52.227.1. ‘Authorization and Consent’ is an affirmative defense that was originally intended to aid government procurement efforts during World War I (Richmond Screw Anchor Co. v. United States, 275 U.S. 331, 345, 48 S.Ct. 194, 72 L.Ed. 303 (1928); Madey v. Duke University, 413 F. Supp.2d 601 (District Court, M.D. North Carolina, 2006). For ‘Authorization and Consent’, two conditions must be met: the activity must be conducted for the government, meaning it is ‘in furtherance and fulfillment of a statement of government policy’ which serves the Government's interest and is for the Government's benefit; and second, the authorization or consent of the Government, which may be express or implied (Madey, 413 F.Supp. 2d 601 (2006)).

147 28 U.S.C. § 1498 (2006) limits the remedies available to patent holder. Patent holders are entitled to fair compensation for the use of the patented invention but no other remedy, such as damages or injunctive relief.

148 See Hughes Aircraft Company v. United States, 29 Fed.Cl. 197 (Fed. Cir. 1993).

149 2011 WL 3875341, Order Grant. Mot. to Dismiss, No. 10–00482, Mar. 16, 2011.

150 Letter, *supra* note 142.

151 Check Hayden, *supra* note 134.

152 Esther Landhuis, *Case Dismissed—NIH Rescues JAX From APPswe Patent Suit*, AlzForum 2011, http://www.alzforum.org/news/community-news/case-dismissed-nih-rescues-jax-appswe-patent-suit (accessed Dec. 23, 2014).

153 Alzheimer's Inst. of America, 2011 WL 3875341; Alzheimer's Inst. Of America, 2013 WL 3305738, *supra* note 6.

154 Daniel M. Skovronsky et al., *In Vivo Detection of Amyloid Plaques in a Mouse Model of Alzheimer's Disease*, 97 PNAS 7609, 7609–7614 (2000).

155 Landhuis, *supra* note 153.

156 2011 WL 3875341, Defendant Avid's Answer, Affirmative Defense, and Countercl. to Plaintiff's Complaint, Jan. 25, 2011.

157 Id.

158 Summary judgment is an exception to the general rule of US patent litigation (indeed, most US civil litigation) that plaintiffs are entitled to a jury trial. However, juries are typically tasked with evaluating disputes of fact while judges evaluate disputes of law. Therefore, a jury trial may be unnecessary if the dispute is entirely over legal conclusions with no genuine dispute over the relevant facts. In such cases, the judge may decide the case summarily as to the legal dispute, with no need for the case to proceed to the jury. For the legal standard for summary judgment and judicial explication of the standard, see Federal Rule of Civil Procedure 56; Celotex Corp. v. Catrett, 477 U.S. 317, *106 S. Ct. 2548*, 91 L. Ed. 2d 265 (1986).

159 Alzheimer's Inst. of America, 2011 WL 3875341, *supra* note 6.

160 Alzheimer's Inst. Of America, 2013 WL 3305738, *supra* note 6.

161 Id.

162 In terms of patent law, individuals who file and prosecute patent applications have ‘a duty of candor and good faith in dealing with the [USPTO], which includes a duty to disclose ...all information known to that individual to be material to patentability’ (Manual of Patent Examining Procedure [hereinafer MPEP] § 1.56– http://www.uspto.gov/web/offices/pac/mpep/documents/appxr_1_56.htm#cfr37s1.56 (accessed Dec. 15, 2014)). The USPTO will not grant patents where there was fraud or if the duty to disclose was ‘violated through bad faith or intentional misconduct’. (MPEP § 1.56).

163 ‘Conception is the ‘formation in the mind of the inventor, of a definite and permanent idea of the complete and operative invention, as it is hereafter to be applied in practice.’ (Hybertech, Inc. v. Monoclonal Antibodies, Inc., 802 F.2d 1367 (Fed. Cir. 1986) quoting *Robinson on Patents* 532 (1890)).

164 See 35 U.S.C. § 116 (2006). Each joint inventor need not have contributed to every feature of the subject matter claimed in the patent, and contributions need not be equal (Sewall v. Walters, 21 F.3d 411, 415 (Fed. Cir. 1994); 35 U.S.C § 116 (2006)). Each need only perform part of the task that produces the invention. A contribution to one claim is enough (SmithKline Diagnostics, Inc. v. Helena Lab.Corp. 859 F.2d 878, 888 (Fed. Cir. 1988).

165 2011 WL 3875341, Defendant Avid's Answer, Jan. 25, 2011.

166 Dalton, *supra* note 102; Landhuis & Strobel, *supra* note 83.

167 Landhuis & Strobel, *supra* note 83.

168 Landhuis & Strobel, *supra* note 83.

169 Dalton, *supra* note 102.

170 Alzheimer's Inst. Of America, 2013 WL 3305738 (citing *Fina Oil etc.*), *supra* note 6, at 2.

171 Alzheimer's Inst. Of America, 2013 WL 3305738, *supra* note 6, at 3.

172 Id.

173 Fla. Admin. Code Ann. R. 6C4–10.012(3)(c), implementing Fla. Stat. § 240.229 (superseded by § 1004.23).

174 Fla. Admin. Code Ann. R. 6C4–10.012(3)(a)(1)).

175 Alzheimer's Inst. of America, 2011 WL 3875341, *supra* note, 6 at 8.

176 *Id.* citing Fla. Admin. Code Ann. R. 6C4–10.012(3)(e)(2)-(3).

177 Alzheimer's Inst. Of America, 2013 WL 3305738, *supra* note 6, at 5.

178 Id.

179 Savage, J. stated, ‘there is ample evidence to support a finding that at the time he signed the May 4, 1992 letter, Newkome had no knowledge of the discovery of the Swedish mutation invention and consequently could not have known that it had been discovered while Mullan was employed at USF. He believed that Mullan and Hardy had not yet begun any new research at USF. Prior to that time, neither Mullan nor Hardy had disclosed that they had done work or made any discoveries while at USF. Nor did Mullan, Hardy or Sexton reveal anything about any such work at the meeting where he signed the letter. Mullan told Newkome at the meeting that he had been unable to do any new work because the laboratories were not ready… The message conveyed, whether intentionally or not, was that work at USF had not yet started’. Alzheimer's Inst. Of America, 2013 WL 3305738, *supra* note 6.

180 Alzheimer's Inst. Of America, 2013 WL 3305738, *supra* note 6, at 10.

181 Landhuis, *supra* note 54.

182 Landhuis, *supra* note 54.

183 Alzheimers Inst. of America, Inc. v. Avid RadioPharmaceuticals, Nos. 2013–1544 (Fed. Cir. May 16, 2014).

184 Alzheimer's Inst. of America, Nos. 2013–1544 (2014). The Order dismissing suit due against the Oklahoma Medical Research Foundation and Comentis due to AIA's lack of standing was entered on Dec. 17, 2012. The case against Elan and remaining co-defendants was decided with prejudice in favor of the defendants on Aug.3, 2012 based on the jury decision that AIA lacked standing to bring the suit. The magistrate granted summary judgment and denied AIA's request to stay the case through its ongoing appeals against Avid Pharmaceuticals and the University of Pennsylvania. It is likely that litigation against the other defendants, including Myriad Genetics, settled, but the outcomes are not clear.

185 Landhuis, *supra* note 54. AnaSpec, Inc., Fremont, California; Immuno-Biological Laboratories, Inc., Minneapolis, Minnesota; Phoenix Pharmaceuticals, Inc., Burlingame, California; and American Peptide Company, Inc., Sunnyvale, California.

186 Landhuis, *supra* note 54.

187 Association for Molecular Pathology v. Myriad Genetics, *supra* note 5, at 1.

188 Mayo Collaborative Services v. Prometheus Lab, *supra* note 5.

189 In Re: *BRCA1* and *BRCA2*-based Hereditary Cancer Test Patent Litigation 2014–1361, 1366 (C.A.F.C., 2014).

190 Elan Pharmaceuticals, Inc. and Athena Neurosciences, Inc. v. Mayo Foundation for Medical Education and Research, *supra* note 104.

191 U.S. Patent No. 5455,169, (filed Jun. 4, 1992).

192 Brigid Hogan, et al., Manipulating the Mouse Embryo (1986).

193 Jon W. Gordon et al., *Genetic Transformation of Mouse Embryos by Microinjection of Purified DNA*, 77 Proc. Natl. Acad. Sci. USA 7380 (1980).

194 Ralph L. Brinster et al., *Somatic Expression of Herpes Thymidine Kinase in Mice Following Injection of a Fusion Gene into Eggs*, 27 Cell 223 (1981).

195 Timothy A. Stewart et al., *Spontaneous Mammary Adenocarcinomas in Transgenic Mice That Carry and Express MTV/myc Fusion Genes*, 38 Cell 627, 627–37 (1984).

196 Jennifer Hogan et al., *Building a Knowledge Base for Systems Pathology*, 9 Br. Bioinform. 518 (2008); Hogan, *supra* note 194.

197 Christine Sturchler-Pierrat et al., *Two Amyloid Precursor Protein Transgenic Mouse Models with Alzheimer Disease-like Pathology*, 94 Proc. Natl. Acad. Sci. USA 13287, 13287–92 (1997). Currently available mouse models and their relative utility are discussed in Jeannie Chin, *Selecting a Mouse Model of Alzheimer's Disease* in 670 Alzheimer's Disease and Frontotemporal Dementia, Methods in Molecular Biology 169–89 (Erik D. Roberson ed., 2011). The article lists 10 APP mouse models, with five accompanying papers published between 1995 and 1999 and 12 papers in the 2000s. Further publications on mouse models for Alzheimer's disease also emerged from 1997 onwards, including from more researchers at The Scripps Institute and Novartis.

198 Dora Games, *Alzheimer-type Neuropathology in Transgenic Mice Overexpressing V717F Beta-amyloid Precursor Protein*, 373 Nature 523, 523–27 (1995).

199 Tanzi & Parson, *supra* note 19, at 144–145.

200 Karen Duff & John Hardy, *Mouse Model Made*, 373 Nature 476, 476–77 (1995).

201 Id.

202 Tanzi & Parson, *supra* note 19, at 219–222.

203 Hsiao et al., *supra* note 92.

204 Chin, *supra* note 195.

205 Tanzi & Parson, *supra* note 19, at 219–222.

206 35 U.S.C. § 154 (amended by Uruguay Round Agreement Act, Pub. L. No. 103–465, 108 Stat. 4809 (1994)).

207 Robin Feldman, *Intellectual Property Wrongs*, 18 Stan. J.L. Bus. & Fin. 250, 264 (2013).

208 Mark A. Lemley, *Rational Ignorance at the Patent Office*, 95 Nw. U. L. Rev. 1495 (2001).

209 President's Council of Economic Advisers, the National Economic Council, and the Office of Science & Technology Policy. Patent Assertion and US Innovation (June 2013), http://www.whitehouse.gov/sites/default/files/docs/patent_report.pdf (accessed Dec. 16, 2014).

210 Id.

211 Leahy-Smith America Invents Act, Pub. L. 112–29, § 6, 125 Stat. 284 (2011).

212 President's Council, *supra* note 207, at 1.

213 Id.

214 President's Council, *supra* note 210, at 6. As an example of the high cost of litigation, Eliot Marshall documented that the University of California, San Francisco (UCSF) spent ${\$ }$12M and Eli Lilly and Company spent ${\$ }$18.5M in 1997 in a patent dispute over the insulin gene (Eliot Marshall, *Court Takes a Narrow View of UC's Claims*, 277 Science 1029 (1997)). That case ended at the Court of Appeals for the Federal Circuit (The Regents of the University of California v. Eli Lilly and Company, 119 F.3d 1559 (Fed. Cir. 1997).

215 Another example described in Blischak, M. P. IP Litigator Jan./Feb., 45–47 (2007) is Housey Pharmaceuticals Inc. v. Abbott Pharmaceutical Inc., et al., No. 01–401 SLR (D.Del). Nov. 27, 2002 aff'd, 366 F.3d 1348 (Fed. Cir. 2004), *cert. denied*, 543 U.S. 1096 (2005) in which the plaintiff patent owner sued nearly 30 innovative companies in bio-pharmaceutical development in 2001 in the District of Delaware for infringement of several patents broadly related to cell-based assay technology. The plaintiff lost on the construction of claims that would have led to a finding of both invalidity and non-infringement.

216 Leahy-Smith America Invents Act, *supra* note 209.

217 Innovation Act, H.R. 3309, 113th Cong. (2013), Patent Transparency and Improvements Act, S. 1720, 113th Cong. (2013), Patent Quality Improvement Act, S. 866, 113th Cong. (2013); Patent Quality Improvement Act, S. 866, 113th Cong. (2013); Patent Abuse Reduction Act, S. 1013, 113th Cong. (2013); Patent Litigation Integrity Act, S. 1612, 113th Cong. (2013); Transparency in Assertion of Patents Act, S. 2049, 113th Cong. (2013); Patent Fee Integrity Act, S. 2146, 113th Cong. (2013); Trade Protection Not Troll Protection Act, H.R. 4763, 113th Cong. (2013); Demand Letter Transparency Act, H.R. 3540, 113th Cong. (2013); Innovation Protection Act, H.R. 3309, 113th Cong. (2013); Patent Litigation and Innovation Act, H.R. 2639,113th Cong. (2013); SHIELD Act, H.R. 845, 113th Cong. (2013); Stopping the Offensive Use of Patents Act (STOP Act), H.R. 2766, 113th Cong. (2013); End Anonymous Patents Act, H.R. 2024, 113th Cong. (2013). For summary see Patent Progress. Patent Progress's Guide to Federal Patent Reform Legislation. See also Patent Progress, Patent Progress's Guide to Federal Patent Reform Legislation, http://www.patentprogress.org/patent-progress-legislation-guides/patent-progresss-guide-patent-reform-legislation/ (accessed Dec. 18, 2014).

218 Joe Mullin*, How the Patent Trolls Won in Congress*. Ars Technica, May 23, 2014, http://arstechnica.com/tech-policy/2014/05/how-the-patent-trolls-won-in-congress/ (accessed Dec. 24, 2014).

219 H.R. 3309, *supra* note 215.

220 Targeting Rogue and Opaque Letters Act, H.R. 4450, 113th Cong. (2014).

221 15 U.S. Code § 41.

222 Daniel Nazer. *The TROL Act is not Enough to to Stop Patent Trolls*, Electronic Frontier Foundation, Jul. 11, 2014, https://www.eff.org/deeplinks/2014/07/when-it-comes-stopping-patent-trolls-trol-act-not-enough (accessed Dec. 24, 2014).

223 Octane Fitness v. ICON Health & Fitness, Inc.*, 134 S. Ct. 981* (2014) (on fee-shifting); eBay Inc. v. MercExchange*, LL*, 126 S. Ct. 1837 (2006) (on loosening of rules for injunctions).

224 Leahy-Smith America Invents Act, *supra* note 209, at § 299.

225 See Bell Atlantic Corp. v. Twombly*, 127 S. Ct. 1955* (2007) and Ashcroft v. Iqbal*, 129 S. Ct. 1937* (2009).

226 Dennis Crouch, *Federal Circuit Supports Bare-Bones Patent Complaints*, PatentlyO, Apr. 23, 2013, http://patentlyo.com/patent/2013/04/federal-circuit-supports-bare-bones-patent-complaints.html (accessed Dec. 24, 2014).

227 Leahy-Smith America Invents Act, *supra* note 209, at § 6.

228 Art.100, E.P.C. (European Patent Convention).

229 Kristina V.J Cornish. *European Patent Oppositions and Biotechnology*. 18 Nat. Biotechnol. 899, 899–900 (2000).

230 Madey, 307 F.3d 1351 (2002) *supra* note 137.

231 Intellectual Property Laws Amendment (Raising the Bar) Act 2012, no. 35, sched. 2 (Austl.)

232 35 U.S.C. §§ 1–376.

233 Riesenfeld, S. A. *University of Pennsylvania Law Review* 102, 291–322 (1954).

234 Patents Act 1990, No. 83, s. 133; s. 163 (Austl.)

235 Productivity Commission Australian Government, *Compulsory Licensing of Patents*, May 27, 2013, http://www.pc.gov.au/projects/inquiry/patents/report (accessed Dec. 24, 2014). But see Jane Nielsen, Dianne Nicol & John Liddicoat, *Centre for Law and Genetics, Submission No. 3 Productivity Commission, Compulsory Licensing of Patents Inquiry*, Sept. 26,2012, at 13. And Tyrone Berger, *Compulsory Licensing of Patents in Australia: reforming the Landscape of Fencing Us In?* 39 Monash U. L. Rev. 915 (2014), http://ssrn.com/abstract=2352316 (accessed Dec. 24, 2014).

236 See LiLan Ren, *Comparison of 28 U.S.C. 1498(A) and Foreign Statutes and an Analysis of 1498(A)'s Compliance with TRIPS (comparing compulsory licensing provisions across jurisdictions)*, 41 Hous. L. Rev. 1659 (2004–2005).

237 See Morrison Forrester, *The Government`s Patent Policy: The Bayh-Dole Act and Authorization and Consent*, Oct. 6, 2002, http://www.mofo.com/resources/news/2002/10/the-governments-patent-policy-the-bayhdole-act-a__#8 (accessed Dec. 24, 2014) (comparing the history and operation of these two acts).

238 35 U.S. Code § 202(c)(4) (Retention of Rights).

239 It refers not only to the US Government but also to ‘a responsible applicant or applicants’.

240 35 U.S.C. § 203 (march-in rights ). These may be granted in the following circumstances(§ 203(a): ‘(1) action is necessary because the contractor or assignee has not taken, or is not expected to take within a reasonable time, effective steps to achieve practical application of the subject invention in such field of use; (2) action is necessary to alleviate health or safety needs which are not reasonably satisfied by the contractor, assignee, or their licensees; (3) action is necessary to meet requirements for public use specified by Federal regulations and such requirements are not reasonably satisfied by the contractor, assignee, or licensees; or (4) action is necessary because the agreement required by section 204 has not been obtained or waived or because a licensee of the exclusive right to use or sell any subject invention in the US is in breach of its agreement obtained pursuant to section 204’.

241 John Conloy, *Government Refuses to March-In Under Bayh-Dole – Again*, Jan. 18, 2011 Genomics Law Report, http://www.genomicslawreport.com/index.php/2011/01/18/government-refuses-to-march-in-under-bayh-dole-again/ (accessed Mar. 25, 2015).

242 U.S. Const. amend V.

243 The wording for and procedures relevant for granting *Authorization and Consent* are outlined in the *Federal Acquisition Regulations* 52.227–1 and 27.2012–2, respectively. *Authorization and Consent* is most commonly used in defense R&D contracts, however, it has been employed for the use and development of research tools.

244 Two international consortia—the International Knockout Mouse Consortium and the International Mouse Phenotyping Consortium —are constructing standardized mouse models with phenotyping data to study gene function. These consortia distribute through established repositories in North America, Europe, and other developed regions. Underlying patents that cover reagents and methods are difficult to identify in this complex environment the use of authorization and consent to protect the US entities involved in the Consortia. Amrita Mishra & Tania Bubela. *Legal Agreements and the Governance of the Research Commons: Lessons from Materials Sharing in Mouse Genomics.* 18 Omics 254 (2014).

245 Association for Molecular Pathology v. Myriad Genetics, *133 S. Ct. 2107* (2013), *supra* note 5.

246 D'Arcy v. Myriad Genetics Inc., (2014) FCAFC 115.

247 Id.

248 Prometheus Lab., 566 U.S. 10 (2012), *supra* note 5.

249 Bilski v. Kappos, 561 U.S. 593 (2010), *supra* note 5.

250 Alice Corp. Pty. Ltd. v. CLS Bank Intern., *134 S. Ct. 2347* (2014), *supra* note 5.

251 D'Arcy v. Myriad Genetics, Inc., *supra* note 244.

252 US Patent and Trademark Office, 2014 Interim Guidance on Patent Subject Matter Eligibility, http://www.gpo.gov/fdsys/pkg/FR-2014-12-16/pdf/2014-29414.pdf (accessed Mar. 25, 2015).

253 Octane Fitness*, 134 S. Ct. 1749* (2014), *supra* note 221.

254 Randall R. Rader et al., *Make Patent Trolls Pay in Court*, N.Y. Times, Jun. 4, 2013, at A25.

255 Jonathan Masur & Anup Malani, *Raising the Stakes in Patent Cases*, 101 Geo. L.J. 637 (2013).

256 Magistrate Judge Faith Angell scheduled a Dec. 2, 2014 hearing. There were 21 attorneys copied on the order scheduling the hearing (Lex Machina, *supra* note 80) .

257 Mark A. Lemley, *Are Universities Patent Trolls?*, 18 Fordham Intell. Prop. Media & Ent. L.J. 611 (2008).

258 *In the Public Interest: Nine Points to Consider in Licensing University Technology.* Mar. 6, 2007, http://www.autm.net/Nine_Points_to_Consider1.htm (accessed Dec. 24, 2014). Note that while the Association of University Technology Managers (AUTM) endorses the Nine Points, they originated from meetings at Stanford University. Note, however, that AUTM is in the process of reconsidering its policy recommendation on licensing to NPEs and AUTM opposed the Innovation Act, *supra* note 215.

259 Bayh-Dole Patent and Trademark Amendments Act of 1980, § 203, codified at 35 U.S.C. §§ 200–12.

260 William W. Goodrich, Jr., *Past Performance as an Evaluation Factor in Public Contract Source Selection*, 47 Am. U.L. Rev. 1539 (1998).

261 Joe Fore et al., *The Effects of Business Practices, Licensing, and Intellectual Property on Development and Dissemination of the Polymerase Chain Reaction: Case Study*, 1 J. Biomed. Disc. Collab. 1, 1, 17 (2006).

262 PatentInsurance, *Intellectual Property Insurance Services Corporation*, 2014, http://www.patentinsurance.com/ (accessed Dec. 14, 2014).

263 Landhuis & Strobel, *supra* note 83.

264 Arthur J. Shannon et al., *Perrigo Company plc Completes Acquisition of Elan Corporation, plc*, (2014), http://perrigo.investorroom.com/2013-12-18-Perrigo-Company-plc-Completes-Acquisition-of-Elan-Corporation-plc (accessed Dec. 18, 2014).

265 Alzheimer's Institute of America Inc., *Supporting Brain Research Worldwide*, 2009, http://www.alzheimersinstitute.com/index.php (accessed Dec. 15, 2014).

266 AIA Foundation, Alzheimer's Institute of America, 2014, http://aiafoundation.org/ (accessed Dec. 18, 2014).

267 UCL, John Hardy, Reta Lila Weston Institute, 2014, http://www.ucl.ac.uk/rlweston-inst/people/john (accessed Dec. 18, 2014).

268 All publication and citation data from Scopus (search conducted 10/7/2014).

269 John Reid Blackwell, *Star Scientific Changes Name and Moves Offices to Florida*, Richmond Times-Dispatch, June 3, 2014. http://www.richmond.com/business/local/article_4f447fc4-eb2a-11e3-89c1-001a4bcf6878.html (accessed Apr. 1, 2015).

270 Michael Pollick, *Roskamp Taking Over Promising Drug Firm*, Herald-Tribune, Apr. 8, 2014. http://www.heraldtribune.com/article/20140408/ARTICLE/140409674 (accessed Apr. 1, 2015).

271 Id.

272 Updated Mar., 2014.

273 Alzheimer's Inst. of America, Inc. v. Avid Radiopharmaceuticals, Memorandum Opinion (E.d., Pa. Mar. 30, 2015).

274 Alzheimer's Inst. of America, Inc. v. Avid Radiopharmaceuticals, Memorandum Opinion (E.d., Pa. Mar. 30, 2015).

275 Octane Fitness*, supra* note 223.

276 *Id. supra* note 1 at p. 1756.

277 AIA v. Avid, *supra* note 1 at p. 5.

278 *Id.* at p. 6.

279 *Id.* at p. *6.*

